# Jmjd3/IRF4 axis aggravates myeloid fibroblast activation and m2 macrophage to myofibroblast transition in renal fibrosis

**DOI:** 10.3389/fimmu.2022.978262

**Published:** 2022-09-08

**Authors:** Hua Liang, Benquan Liu, Ying Gao, Jiayi Nie, Shuyun Feng, Wenqiang Yu, Shihong Wen, Xi Su

**Affiliations:** ^1^Department of Anesthesiology, Foshan Women and Children Hospital, Foshan, China; ^2^Department of Anesthesiology, Affiliated Foshan Women and Children Hospital of Southern Medical University, Foshan, China; ^3^Department of Anesthesiology, The First People’s Hospital of Foshan, Foshan, China; ^4^Department of Anesthesiology, The First Affiliated Hospital of Sun Yat-Sen University, Guangzhou, China; ^5^Department of Paediatrics, Foshan Women and Children Hospital, Foshan, China

**Keywords:** Jmjd3, IRF4, renal fibrosis, fibroblast, macrophage

## Abstract

Renal fibrosis commonly occurs in the process of chronic kidney diseases. Here, we explored the role of Jumonji domain containing 3 (Jmjd3)/interferon regulatory factor 4 (IRF4) axis in activation of myeloid fibroblasts and transition of M2 macrophages into myofibroblasts transition (M2MMT) in kidney fibrosis. In mice, Jmjd3 and IRF4 were highly induced in interstitial cells of kidneys with folic acid or obstructive injury. Jmjd3 deletion in myeloid cells or Jmjd3 inhibitor reduced the levels of IRF4 in injured kidneys. Myeloid Jmjd3 depletion impaired bone marrow-derived fibroblasts activation and M2MMT in folic acid or obstructive nephropathy, resulting in reduction of extracellular matrix (ECM) proteins expression, myofibroblasts formation and renal fibrosis progression. Pharmacological inhibition of Jmjd3 also prevented myeloid fibroblasts activation, M2MMT, and kidney fibrosis development in folic acid nephropathy. Furthermore, IRF4 disruption inhibited myeloid myofibroblasts accumulation, M2MMT, ECM proteins accumulation, and showed milder fibrotic response in obstructed kidneys. Bone marrow transplantation experiment showed that wild-type mice received IRF4^-/-^ bone marrow cells presented less myeloid fibroblasts activation in injured kidneys and exhibited much less kidney fibrosis after unilateral ureteral obstruction. Myeloid Jmjd3 deletion or Jmjd3 inhibitor attenuated expressions of IRF4, α-smooth muscle actin and fibronectin and impeded M2MMT in cultured monocytes exposed to IL-4. Conversely, overexpression IRF4 abrogated the effect of myeloid Jmjd3 deletion on M2MMT. Thus, Jmjd3/IRF4 signaling has a crucial role in myeloid fibroblasts activation, M2 macrophages to myofibroblasts transition, extracellular matrix protein deposition, and kidney fibrosis progression.

## Introduction

Chronic kidney disease (CKD) is referred to as an emergent worldwide health issue that imposes a substantial challenge and financial burden to communities and individuals (1). Renal fibrosis, featured with abnormal production of extracellular matrix (ECM) proteins, is recognized as the common mechanism whereby progressive renal injuries of diverse etiologies lead to progressive CKD and end-stage renal disease (ESRD) (2, 3). Availability of CKD therapeutic option is still limited in clinical. Patients nearing ESRD requires a kidney transplant or dialysis to survive (4, 5). As such, novel insight into this devastating disorder is imperative for anti-fibrotic treatment developments.

Myofibroblasts are considered as the major ECM-producing cells and the central mediators of renal fibrosis (6, 7). Recently, strong evidences have documented that the transformation of infiltrating bone marrow–derived cells into myofibroblasts (8–10) and M2 macrophages to myofibroblasts transition (M2MMT) exert a pivotal role in renal fibrosis (11–14). However, the molecular mechanisms that help myeloid fibroblasts activation and M2MMT remain largely to be elucidated. IL-4-Janus kinase 3 (JAK3)-signal transducer and activator of transcription 6 (STAT6) axis belongs to a classical pro-fibrotic Th2 cytokines signaling pathway, which is deeply linked to myeloid fibroblasts activation and macrophage M2 polarization (15). Our study has demonstrated that IL-4 receptor α (IL-4Rα)/STAT6 axis play an important role in renal fibrosis (16). Loss of IL-4Rα inhibits STAT6 activation, suppresses bone marrow-derived fibroblasts activation, impairs macrophage M2 polarization, and attenuates renal fibrosis (16). Additionally, JAK3/STAT6 signaling stimulates bone marrow-derived fibroblasts activation, trigger macrophage to a pro-fibrotic M2-like phenotype in renal fibrosis process (15, 17). In vitro, Th2 cytokines drives bone marrow-derived monocytes to a myofibroblast phenotype through JAK3/STAT6 signaling pathway (15). Nevertheless, the molecular signaling mechanism underlying myeloid fibroblasts activation and macrophage M2 polarization in injured kidneys requires further investigation.

Jumonji domain containing 3 (Jmjd3), a jumonji domain-containing histone demethylase, is able to demethylate histone and activate gene expression (18, 19). As a direct downstream target of IL-4/JAK3/STAT6 axis, Jmjd3 has a central regulatory role in bone marrow macrophage differentiation (20, 21). Interferon regulatory factor 4 (IRF4), the fourth member of the IRF family, plays a critical role in regulating the differentiation of lymphoid, myeloid, and dendritic cells (22, 23). IRF4 is the direct target gene of Jmjd3 and triggers M2 macrophage polarization (24, 25). Moreover, Jmjd3/IRF4 axis has been identified in host responses against helminth infection (21) and in granulocyte macrophage colony-stimulating factor–driven pathway (26–28). However, the role of Jmjd3/IRF4 axis in fibrotic kidneys remains unclear.

Mounting evidences show that inhibition of Jmjd3 or IRF4 deficiency protects against fibrotic process (23, 24, 29–33). Although a study showed that disruption of Jmjd3 from interstitial fibroblasts exacerbates kidney fibrosis (34), the effect of myeloid-specific deletion of Jmjd3 on renal fibrosis progression remains undefined. Given that Jmjd3 is a direct downstream target of IL-4/STAT6 signaling and the essential role of Jmjd3/IRF4 axis in bone marrow-derived macrophages differentiation (20, 21), it is tempting to speculate that targeted inhibition Jmjd3/IRF4 signaling suppresses the activation of myeloid fibroblasts and M2MMT in kidney fibrosis development.

Here, we provided experimental evidence that Jmjd3/IRF4 axis plays an important role in myeloid fibroblast activation and M2MMT in folic acid (FA) nephropathy or obstructive nephropathy. We show that Jmjd3 depletion in myeloid cells (Jmjd3^flox^/^flox^ Lyz2Cre^+^) or IRF4 disruption impairs myeloid fibroblasts activation, inhibits M2MMT, and attenuates renal fibrosis progression.

## Materials and methods

### Animals and models

Jmjd3^flox^/^flox^ mice, Lyz2-Cre mice, and IRF4 knockout mice were purchased from Shanghai Model Organisms Center, Inc. All mice for target gene deletion were on a C57BL/6J background. To obtain Lyz2-Cre^+^ Jmjd3^flox^/^flox^ mice (myeloid Jmjd3^-/-^), Jmjd3^flox^/^flox^ mice were crossed with Lyz2-Cre mice. Male mice (8 weeks old, weighing 20 g-25 g) were used in this experiment. All experiments conformed to the Guidelines of the National Institutes of Health (NIH publication, No. 8023) on the ethical use of animals. All animals handling and surgical procedures were according to the Animal Ethics Committee of Sun Yat-sen University (No. 2017-692). For FA-induced renal fibrosis, mice were treated with a single intraperitoneal injection of FA at 250 mg/kg. For pharmacological inhibition of Jmjd3, mice were treated with GSKJ4 at daily dose of 10 mg/kg via intraperitoneal injection for 14 days. For UUO surgery, the left ureter was ligated with 4-0 silk suture as previously described (16). Kidney tissues were collected at 14 days for next experiments.

### Renal histology

Kidney tissues were fixed in 10% paraformaldehyde and embedded in paraffin blocks. The 4-μm sections were prepared. After deparaffinization, Hematoxylin and eosin (H&E), Sirius red and Masson’s trichrome staining were employed to determine fibrotic injury. A microscope (Olympus Microsystems, Japan) was used to observe sections. The NIS-Elements BR 4.0 software was employed to analyze images (16).

### Immunofluorescence

The frozen kidney tissues were embedded with OCT and cut into 5 μm-thick sections and stored at -80°C until use. After the frozen sections were dried, they were fixed with acetone, rinsed three times with PBS, blocked with goat serum for 1 h, and then incubated with primary antibody (fibronectin antibody and collagen I antibody, Abcam) overnight at 4°C. After washing with PBS, the Alexa Fluor 488 was applied for 1 h. For α-SMA staining, kidney tissues were cut into 4 μm-thick sections after embedding in paraffin, which were subjected to antigen retrieval, washed with PBS, blocked, and incubated overnight with Cy3-conjugated α-SMA antibody (Sigma). Frozen sections were incubated overnight with primary CD45 (Bioscience) or CD11b (Bio-Rad) or CD206 (Bio-Rad) and α-SMA (Abcam) antibodies for double immunofluorescence staining, and then followed by applied with Alexa Fluor 488 or 647. Sections were mounted with DAPI and mounting medium. Images were obtained from fluorescence microscope (Nikon, Japan) and Zeiss confocal microscope (oberkochen, Germany).

### Immunohistochemistry

Kidney tissues were embedded in paraffin, fixed and cut into 4 μm-thick sections for immunohistochemical staining. After antigen retrieval, treated with 3%H2O2, and blocked with serum, sections were incubated with primary antibodies overnight at 4°C. After washing with PBS, sections were incubated sequentially with secondary antibodies and ABC solutions according to ABC kit instructions (Vector Laboratories). Slides were incubated with diaminobenzidine and nuclear was stained with hematoxylin.

### Western blots

Standard procedure for extracting protein in renal tissue was used. BCA protein assay was employed to determine protein concentration. Protein in lysates were loaded equally and separated on SDS-PAGE gel and transferred to PVDF membrane before blocking. After incubation with 5%BSA, the membranes were incubated with primary antibodies and secondary antibodies in sequence. The Jmjd3 antibody, fibronectin antibody, and collagen I antibody were purchased from Abcam corporation. The α-SMA antibody was obtained from Sigma Corporation. The GAPDH antibody and β-actin antibody were purchased from Santa Cruz. ECL reagents were used for detecting chemiluminescence signals.

### Quantitative real-time PCR analysis

Total RNA from kidney tissues was isolated using Trizol (TaKaRa, Shiga, Japan), according to the protocol. The total RNA concentrations of samples were detected by the SYBR® Premix Ex Taq™ II kit (TaKaRa) on an ABI 7500 Real Time PCR System (Applied Biosystems, USA). Then, we used IQ SYBR green super-mix reagent (Bio-Rad) for cDNA synthesis. Subsequently, quantitative real-time PCR was performed in the real-time PCR machine (Bio-Rad). The primers of target genes were as follows: GAPDH forward primer: 5’-CCAATGTGTCCGTCGCGTGGATCT-3’ and reverse primer: 5’-GTTGAAGTCGCAGGAGACAACC-3’; Arg-1 forward primer: 5’-TTATCGAGCGCCTTTCTCAA-3’ and reverse primer: 5’-TGGTCTCTCAGGTCATACTCTGT-3’; FIZZ1 forward primer: 5’-CAATCCCATGGCGTATAAAAGCATC-3’ and reverse primer: 5’- TCATTCT-TAGGACAGTTGGCAGC-3’; TNF-α forward primer: 5’-ATGAGCACAGAAA-GCATGATC-3’ and reverse primer: 5’- TACAGGCTTGTCACTCGAATT-3’; IL-6 forward primer: 5’- ACAAAGCCAGAGTCCTTCAGAGA-3’ and reverse primer: 5’- CTGTTAGGAGAGCATTGGAAATTG-3’. The relative gene expression was calculated by the 2−ΔΔCT method.

### Bone marrow transplantation

The experiment of bone marrow transplantation was employed as previously described (15, 16). Wild-type mice received lethal irradiation. The myeloid cells (5×10^6^) from wild-type or IRF4 knockout mice were then transferred to the mice via tail vein injection. The mice that received bone marrow cells were allowed to recovery for eight weeks.

### Bone marrow monocytes culture

Bone marrow monocytes were obtained from wild-type or IRF4 deficiency mice. The monocytes were cultured as previously described (16). The cells were isolated from the femur and tibia of mice (8 weeks old) and cultured in RPMI medium containing 10% FBS, 10% L929-conditioned medium, 1% glutamine, 1% MEM vitamins, and 1% penicillin/streptomycin at 37°C and 5% CO_2_. The cells were starved for 24 hours before IL-4 (50 ng/ml) or GSKJ4 (10 μM) treatment. The bone marrow monocytes with Jmjd3 myeloid deletion were transfected with the overexpression plasmid of IRF4 using Lipofectamine 3000 (Invitrogen, USA) according to the manufacturer’s instruction.

### Statistics

All quantitative data were represented as mean ± SEM. Analysis of variance was employed to analyze statistical differences between groups. Bonferroni procedure was used to compare differences between means. *P* value < 0.05 was considered as the significance threshold.

## Results

### Jmjd3/IRF4 signaling axis is activated in FA or obstructive nephropathy

**Figure 1:**
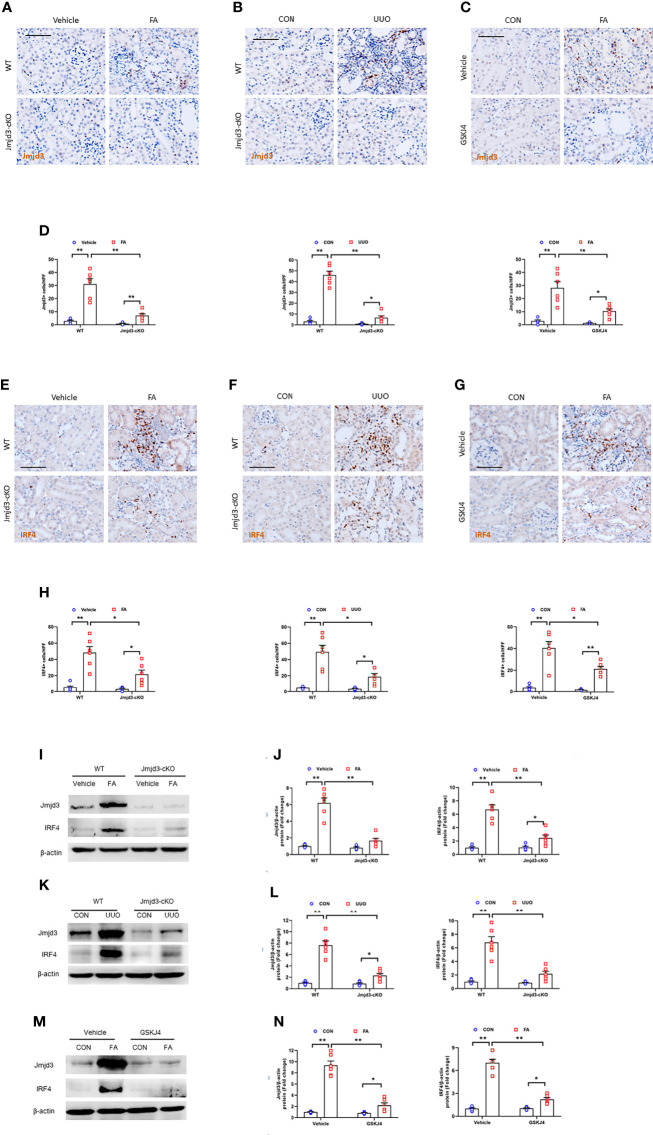
Jmjd3/IRF4 axis is highly activated in kidneys with FA or obstructive injury. **(A–C)** Kidney sections stained for Jmjd3 (brown) at day 14 after FA or UUO treatment. **(D)** Quantitative analysis of Jmjd3^+^ cells. **(E–G)** Representative photomicrographs of kidney sections stained for IRF4 (brown) at day 14 after FA or UUO treatment. **(H)** Quantitative analysis of IRF4^+^ cells. **(I)** Western blot bands of Jmjd3 and IRF4 in kidneys with or without FA injury. **(J)** Quantitative analysis of Jmjd3 and IRF4 protein levels with or without FA injury. **(K)** Western blot bands of Jmjd3 and IRF4 in the kidneys with or without UUO injury. **(L)** Quantitative analysis of Jmjd3 and IRF4 protein levels in the kidneys o with or without UUO injury. **(M)** Western blot bands of Jmjd3 and IRF4 in control or FA-injured kidneys with or without GSKJ4 treatment. **(N)** Quantitative analysis of Jmjd3 and IRF4 protein levels in control or FA-injured kidneys of mice with or without GSKJ4 treatment. *P<0.05 or **P<0.01; n=6; Bar = 50 μm. WT, wild-type; cKO, conditional knockout; HPF, high-power field.

Firstly, we examined the protein levels of Jmjd3 in injured kidneys of mice. We revealed that positive staining cells of Jmjd3 were notably increased in injured kidneys, while myeloid Jmjd3 deletion significantly reduced these cells. The Jmjd3-positive cells were mainly detected in interstitial cells in the kidneys (**Figures 1A–D**). Additionally, IRF4 was highly induced in interstitial cells of the kidneys following folic acid or UUO treatment. In contrast, myeloid Jmjd3 deletion or GSKJ4 treatment abolished IRF4 expression (**Figures 1E–H**). In agreement with the data of immunohistochemical staining, the protein levels of Jmjd3 were considerably increased in kidneys subjected to FA or UUO injury. Given that IRF4 is a direct target gene of Jmjd3, we next examined if myeloid Jmjd3 deletion or Jmjd3 inhibitor has an impact on IRF4 activation. We showed that inhibition of Jmjd3 caused a significant reduction of IRF4 protein level (**Figures 1I–N**). These data indicate that Jmjd3/IRF4 axis is highly activated in FA or obstructive nephropathy.

### Myeloid Jmjd3 deletion attenuates accumulation of myeloid fibroblast and M2MMT in FA-injured kidneys

**Figure 2:**
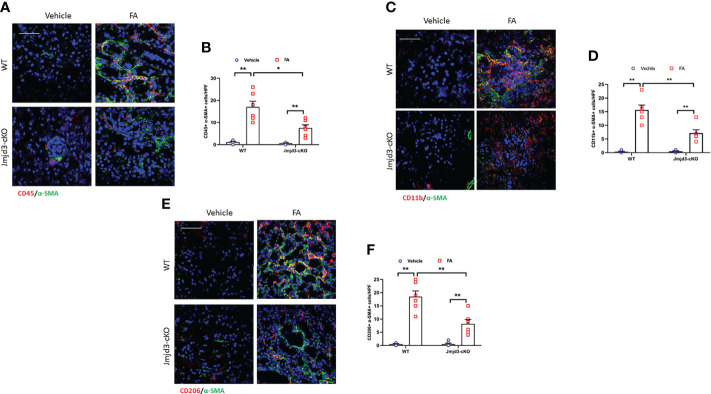
Myeloid Jmjd3 deletion inhibits myeloid myofibroblast accumulation and M2MMT in kidneys with FA injury. **(A)** Kidney sections iwere stained for CD45 (red) and α-SMA (green). **(B)** Quantitative analysis of CD45^+^-α-SMA^+^ cells in kidneys with or without FA injury. **(C)** Kidney sections were stained for CD11b^+^-α-SMA^+^ cells in kidneys with or without FA injury. **(D)** Quantitative analysis of CD11b^+^-α-SMA^+^ cells in kidneys with or without FA injury. **(E)** Kidney sections were stained for CD206 (red) and α-SMA (green). **(F)** Quantitative analysis of CD206^+^-α-SMA^+^ cells in the kidneys with or without FA injury. *P<0.05 or **P<0.01; n=6; Bar = 50 μm. M2MMT=M2 macrophage to myofibroblast transition; WT, wild-type; cKO, conditional knockout; HPF, high-power field.

Bone marrow-derived fibroblasts activation and macrophage M2 polarization are mediated by Th2 cytokines signaling pathway (15, 16, 35). Jmjd3 has been proved to be a downstream target of Th2 cytokines signaling (20, 36). Thus, we next assessed whether Jmjd3 has an impact on in bone marrow-derived fibroblasts activation in FA-injured kidneys of mice. We revealed that CD45^+^-α-SMA^+^ and CD11b^+^-α-SMA^+^ dual-positive cells in the kidneys of control mice following FA stimulation were markedly elevated. Conversely, myeloid Jmjd3 deletion led to a notably decrease of these cells in injured kidneys (**Figures 2A–D**). Jmjd3 can induce macrophage M2 polarization, which depends on IL-4/STAT6 signaling (20, 28). Of note, it has been demonstrated that M2MMT contributes to renal fibrosis progression (14, 37). We next examined if Jmjd3 inhibition impairs M2MMT in the FA-treated kidneys. Our findings showed that targeted inhibition of Jmjd3 markedly reduced CD206^+^-α-SMA^+^ dual-positive cells in injured kidneys of mice (**Figures 2E, F**). In addition, myeloid Jmjd3 deletion ameliorated renal function, alleviated the level of inflammatory cytokines, and resulted in a notably down-regulation of the Arg-1 mRNA and FIZZ1 mRNA in the FA-treated kidneys (**Supplementary Figures 1A–C**). These findings suggest that Jmjd3 in myeloid cells promotes activation of myeloid fibroblasts and differentiation of M2 macrophages into myofibroblasts.

### Myeloid Jmjd3 deletion attenuates fibrosis progression in FA nephropathy

**Figure 3:**
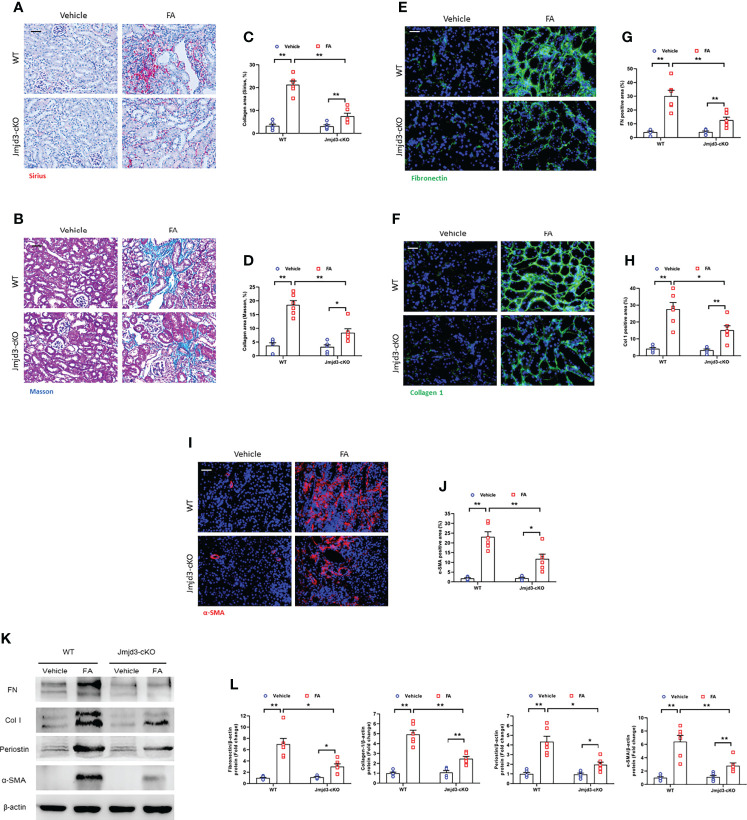
Myeloid Jmjd3 deletion suppresses renal fibrosis, extracellular matrix protein expression, and myofibroblasts accumulation in kidneys with FA injury. **(A, B)** Kidney sections were stained with Sirius red or Masson’s Trichrome reagent. **(C, D)** Quantitative analysis of collagen area of each group. **(E, F)** Kidney sections were stained for fibronectin (green) or collagen I (green). **(G, H)** Quantitative analysis of fibronectin or collagen I-positive area. **(I)** Kidney sections were stained for α-SMA (red). **(J)** Quantitative analysis of α-SMA -positive area in the kidneys. **(K)** Western blots bands of protein expression of fibronectin, collagen I, periostin and α-SMA in the kidneys. **(L)** Quantitative analysis of proteins levels in the kidneys. *P<0.05 or **P<0.01; n=6; Bar = 50 μm. WT, wild-type; cKO, conditional knockout.

Next, the effect of myeloid Jmjd3 deletion on kidney fibrosis of mice with FA nephropathy was investigated. There was severe collagen deposition in FA-injured kidneys compared with controls mice. Conversely, myeloid Jmjd3 deletion led to a substantial reduction of collagen deposition (**Figures 3A–D**). Moreover, FA injury significantly increased the expressions of ECM proteins and α-SMA in FA nephropathy compared with controls group. In contrast, the injured kidneys of mice with myeloid Jmjd3 deletion exhibited lower levels of these proteins (**Figures 3E–L**). These data indicate that myeloid Jmjd3 deletion attenuates the progression of renal fibrosis.

### Myeloid Jmjd3 deletion protects against myeloid fibroblasts activation, M2MMT, and fibrosis progression in obstructive kidneys

**Figure 4:**
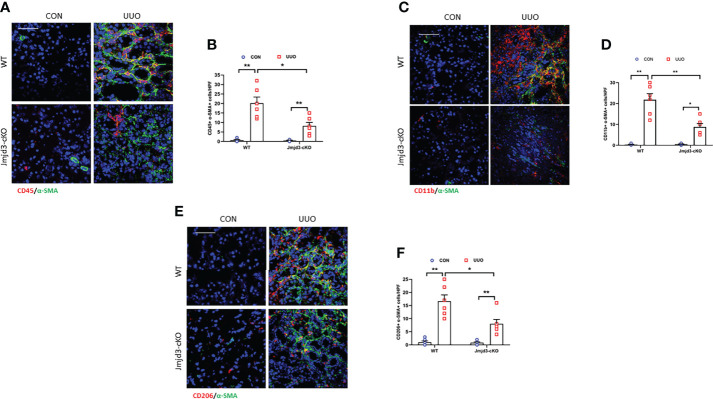
Myeloid Jmjd3 deletion attenuates myeloid fibroblast activation and M2MMT in kidneys with obstructive injury. **(A)** Kidney sections were stained for CD45 (red) and α-SMA (green). **(B)** Quantitative analysis of CD45^+^-α-SMA^+^ cells in the kidneys. **(C)** Kidney sections were stained for CD11b (red) and α-SMA (green). **(D)** Quantitative analysis of CD11b^+^-α-SMA^+^ cells in the kidneys. **(E)** Kidney sections were stained for CD206 (red) and α-SMA (green). **(F)** Quantitative analysis of CD206^+^-α-SMA^+^ cells in the kidneys. *P<0.05 or **P<0.01; n = 6; Bar = 50 μm. M2MMT=M2 macrophage to myofibroblast transition; WT, wild-type; cKO, conditional knockout; HPF, high-power field.

**Figure 5:**
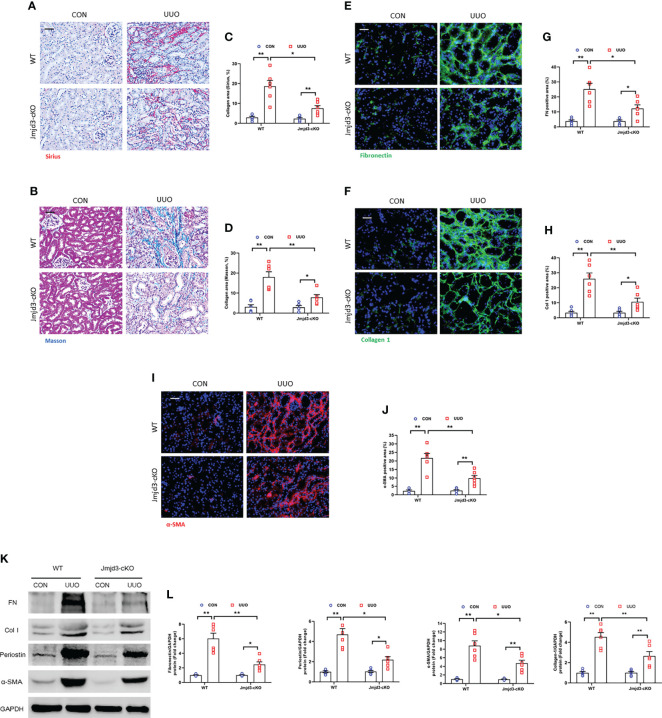
Myeloid Jmjd3 deletion reduces renal fibrosis, extracellular matrix protein expression, and myofibroblasts accumulation in obstructive nephropathy. **(A, B)** Kidney sections were stained with Sirius red or Masson’s Trichrome reagent. **(C, D)** Quantitative analysis of collagen area in the kidneys. **(E, F)** Kidney sections were stained for fibronectin (green) or collagen I (green). **(G, H)** Quantitative analysis of fibronectin or collagen I-positive area. **(I)** Kidney sections were stained for α-SMA (red). **(J)** Quantitative analysis of α-SMA -positive area in the kidneys. **(K)** Representative western blots show protein expression of fibronectin, collagen I, periostin and α-SMA in the kidneys. **(L)** Quantitative analysis of ECM and α-SMA protein levels in the kidneys. *P<0.05 or **P<0.01; n = 6; Bar = 50 μm. WT, wild-type; cKO, conditional knockout.

Our results demonstrated that Jmjd3 has a crucial role in activation of myeloid fibroblasts and M2MMT in FA-injured kidneys. We next examined if myeloid Jmjd3 deletion has an impact on myofibroblasts accumulation, M2MMT, and kidney fibrosis development in obstructive nephropathy. Our findings exhibited that myeloid Jmjd3 deletion profoundly lowered the CD45^+^-α-SMA^+^ and CD11b+-α-SMA^+^ dual positive cells in obstructive nephropathy (**Figures 4A–D**). Our findings also revealed that Jmjd3 inhibition markedly reduced CD206^+^-α-SMA^+^ cells in the kidneys of mice with obstructive nephropathy (**Figures 4E, F**). In agreement with these results, myeloid Jmjd3 deletion resulted in a significant reduction of collagen production and the deposition of ECM proteins and fibroblast activation UUO-treated kidneys (**Figures 5A–L**). These data indicate that Jmjd3 mediates myeloid fibroblasts activation, M2MMT, and fibrosis development in obstructive nephropathy.

### Inhibition of Jmjd3 by GSKJ4 prevents myeloid fibroblast activation and M2MMT, fibrosis progression in FA-injured kidneys

**Figure 6:**
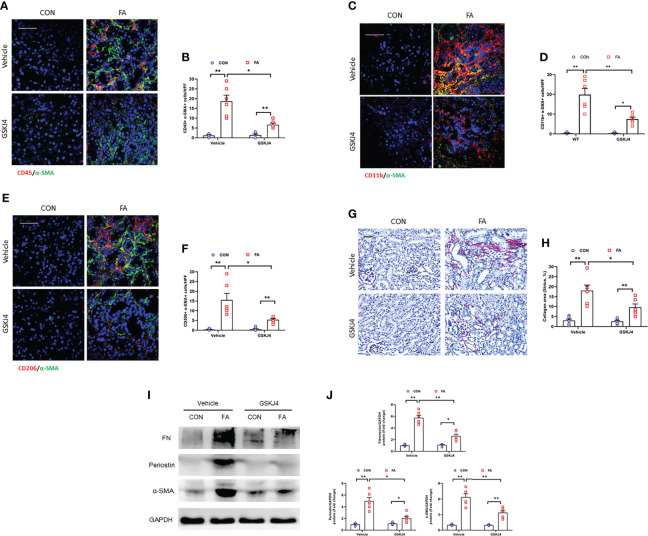
Pharmacological inhibition of Jmjd3 suppresses myeloid fibroblast activation, M2MMT, and renal fibrosis in kidneys with FA injury. **(A)** Kidney sections were stained for CD45 (red), and α-SMA (green). **(B)** Quantitative analysis of CD45^+^-α-SMA^+^ cells. **(C)** Kidney sections were stained for CD11b (red), and α-SMA (green). **(D)** Quantitative analysis of CD11b^+^-α-SMA^+^ cells. **(E)** Kidney sections in each group were stained for CD206 (red) and α-SMA (green). **(F)** Quantitative analysis of CD206^+^-α-SMA^+^ cells in the kidneys. **(G)** Kidney sections were stained with Sirius red. **(H)** Quantitative analysis of collagen area. **(I)** Western blots bands of protein expression of fibronectin, periostin and α-SMA in the kidneys. **(J)** Quantitative analysis of protein levels. *P<0.05 or **P<0.01; n = 6; Bar = 50 μm. HPF, high-power field.

We next explored if inhibition of Jmjd3 by GSKJ4 has an impact on myeloid fibroblasts activation, M2MMT, and kidney fibrosis progression in FA nephropathy. Our results showed that GSKJ4 treatment profoundly decreased the CD45^+^-α-SMA^+^ and CD11b^+^-α-SMA^+^ dual positive cells in kidneys subjected to FA treatment (**Figures 6A–D**). We also revealed that administration of GSKJ4 significantly decreased the CD206^+^-α-SMA^+^ dual positive cells in kidneys treated by FA (**Figures 6E, F**). Consistent with these findings, GSKJ4 treatment resulted in a sharp reduction of collagen deposition and the expression of ECM proteins and α-SMA in FA-injured kidneys (**Figures 6G–J**). These data suggest that pharmacological inhibition of Jmjd3 prevents myofibroblast accumulation, M2MMT, and fibrosis progression in FA nephropathy.

### IRF4 knockout reduces myeloid myofibroblast accumulation, impairs M2MMT in obstructive nephropathy

**Figure 7:**
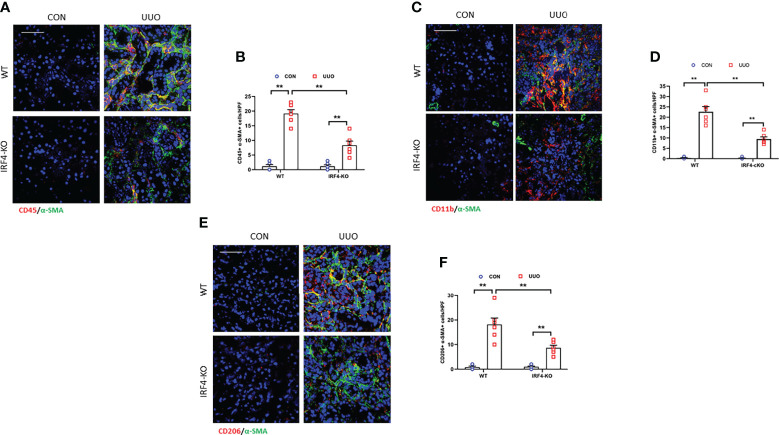
IRF4 deficiency prevents myeloid fibroblast activation and M2MMT in obstructive kidneys. **(A)** Kidney sections were stained for CD45 (red) and α-SMA (green). **(B)** Quantitative analysis of CD45^+^-α-SMA^+^ cells. **(C)** Kidney sections were stained for CD11b (red) and α-SMA (green). **(D)** Quantitative analysis of CD11b^+^-α-SMA^+^cells. **(E)** Kidney sections were stained for CD206 (red) and α-SMA (green). **(F)** Quantitative analysis of CD206^+^-α-SMA^+^ cells in the kidneys. **P<0.01; n = 6; Bar = 50 μm. M2MMT, M2 macrophage to myofibroblast transition; WT, wild-type; KO, knockout; HPF, high-power field.

IRF4 is a target gene of Jmjd3 and responsible for M2 macrophage polarization. It has been proved be closely relevant to fibrotic disorder (21, 38). Thus, we next assessed the role of IRF4 in activation of myeloid fibroblast and differentiation of M2 macrophage to myofibroblast in obstructive nephropathy. Our data show that the CD45^+^-α-SMA^+^ and CD11b^+^-α-SMA^+^ dual positive cells was notably decreased in injured kidneys of IRF4 knockout mice compared with wild-type mice (**Figures 7A–D**). Consistent with these observations, the CD206^+^-α-SMA^+^ cells in obstructed kidneys of wild-type mice displayed a sharp elevation. In comparison, IRF4 disruption considerable lowered the CD206^+^-α-SMA^+^ cells in kidneys with UUO (**Figures 7E, F**). These findings suggest that IRF4 mediates activation of myeloid fibroblast and M2MMT in obstructive nephropathy.

### IRF4 disruption attenuates renal fibrosis development after UUO injury

**Figure 8:**
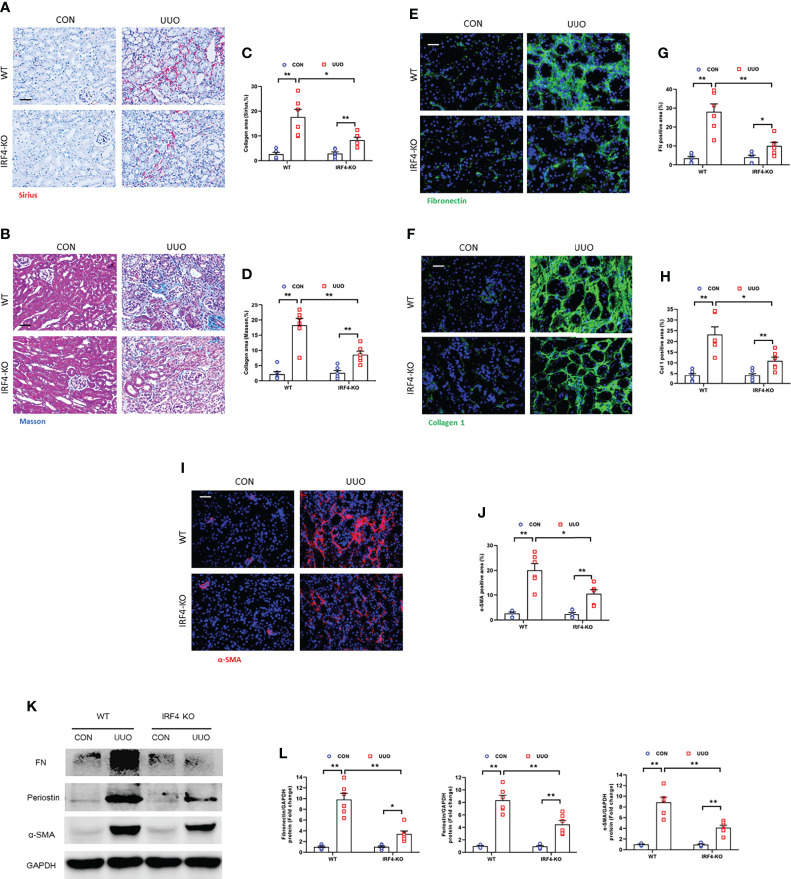
IRF4 deficiency impedes renal fibrosis, extracellular matrix protein expression, and myofibroblasts accumulation in obstructive nephropathy. **(A, B)** Sirius red or Masson’s Trichrome staining for kidney sections. **(C, D)** Quantitative analysis of collagen area in the kidneys. **(E, F)** Kidney sections were stained with fibronectin (green) or collagen I (green). **(G, H)** Quantitative analysis of positive area. **(I)** Kidney sections were stained for α-SMA (red). **(J)** Quantitative analysis of α-SMA -positive area. **(K)** Western blots bands of protein expression of fibronectin, periostin and α-SMA. **(L)** Quantitative analysis of proteins levels in the kidneys. *P<0.05 or **P<0.01; n = 6; Bar = 50 μm. WT, wild-type; KO, knockout.

We next examined the effect of genetic ablation of IRF4 on renal fibrosis induced by UUO injury. We revealed that IRF4 deficiency significantly reduced collagen deposition area in UUO-injured kidneys (**Figures 8A–D**). Moreover, IRF4 deficiency led to a significant reduction of ECM proteins and inhibits fibroblasts activation in kidneys with UUO injury (**Figures 8E–L**). These data suggest that IRF4 knockout attenuates collagen deposition and ECM protein production in kidney fibrosis induced by UUO.

### IRF4 ablation in myeloid cells inhibits myeloid fibroblasts accumulation, M2MMT, and kidney fibrosis progression

**Figure 9:**
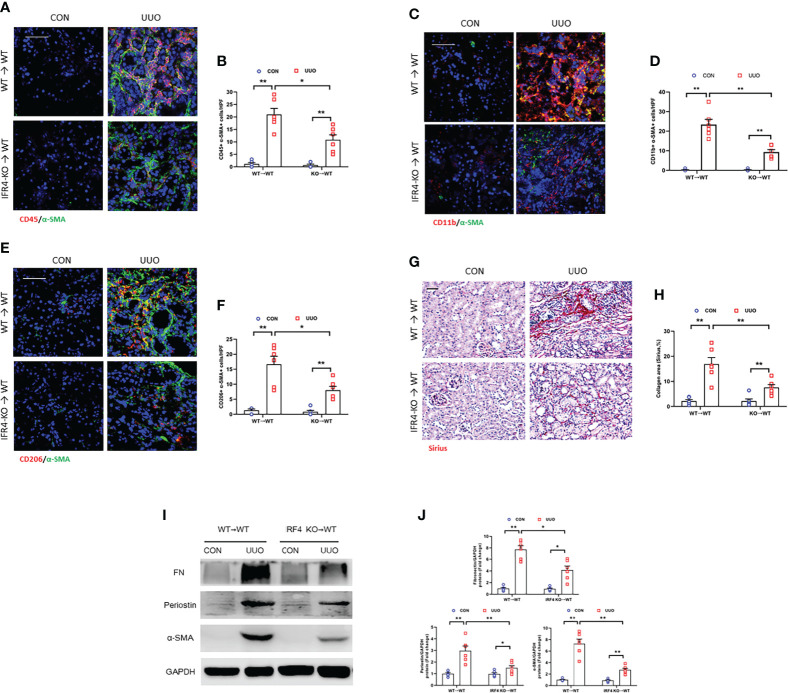
IRF4 deletion in myeloid cells inhibits myeloid fibroblast activation, M2MMT, and kidney fibrosis. **(A)** Kidney sections from WT→WT and IRF4-KO→WT mice after UUO treatment were stained for CD45 (red), and α-SMA (green). **(B)** Quantitative analysis of CD45^+^-α-SMA^+^ cells. **(C)** Kidney sections from WT→WT and IRF4-KO→WT mice after UUO treatment were stained for CD11b (red), and α-SMA (green). **(D)** Quantitative analysis of CD11b^+^-α-SMA^+^ cells. **(E)** Kidney sections from WT→WT and IRF4-KO→WT mice after UUO treatment were stained for CD206 (red) and α-SMA (green). **(F)** Quantitative analysis of CD206^+^-α-SMA^+^ cells. **(G)** Kidney sections from WT→WT and IRF4-KO→WT mice after UUO treatment were stained with Sirius red. **(H)** Quantitative analysis of collagen area. **(I)** Western blots bands of protein expressions in the kidneys. **(J)** Quantitative analysis of protein levels. *P<0.05 or **P<0.01; n = 6; Bar = 50 μm. WT, wild-type; KO, knockout; HPF, high-power field.

Evidences show that myeloid myofibroblasts significantly contributes to renal fibrosis. Also, bone marrow-derived fibroblasts are originated from monocytes via macrophage M2 polarization (15, 16). In order to certify the role of IRF4 in myeloid cells in the progression of kidney fibrogenesis, bone marrow transplantation experiment was employed. The chimeric mice were received unilateral ureteral ligation. There was a sharp decrease of CD45^+^-α-SMA^+^ and CD11b^+^-α-SMA^+^ dual positive cells in UUO kidneys of WT mice engrafted with IRF4^-/-^ myeloid cells (KO→WT) compared with WT mice engrafted with IRF4^+/+^ myeloid cells (WT→WT) (**Figures 9A–D**). WT mice transplanted with IRF4^-/-^ bone marrow cells (KO→WT) presented lower number of CD206^+^-α-SMA^+^ myeloid myofibroblasts in kidneys with UUO (**Figures 9E, F**). IRF4-^+/+^ mice engrafted with IRF4^-/-^ bone marrow cells presented milder kidney fibrogenesis (**Figures 9G–J**). These results suggest that IRF4 signaling in myeloid cells exerts a pivotal role in the activation of bone marrow-derived fibroblasts, M2MMT, and progressive renal fibrosis. These data strengthen the point that bone marrow-derived fibroblasts are originated from monocytes via macrophage M2 polarization, which is modulated by IRF4.

### Jmjd3/IRF4 axis is activated in bone marrow–derived monocytes

**Figure 10:**
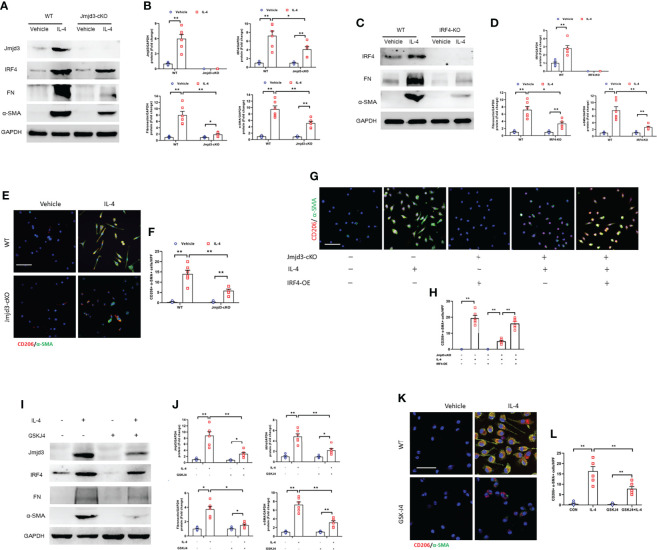
Jmjd3/IRF4 axis is highly activated in cultured monocytes. **(A)** Western blots bands of protein expression of Jmjd3, IRF4, fibronectin, and α-SMA in cultured monocytes. **(B)** Quantitative analysis of Jmjd3, IRF4, fibronectin, and α-SMA protein levels. **(C)** Western blots bands of protein expression of IRF4, fibronectin, and α-SMA in cultured monocytes. **(D)** Quantitative analysis of IRF4, fibronectin, and α-SMA protein levels. **(E)** Monocytes of WT or Jmjd3-cKO mice were stained for CD206 (red) and α-SMA (green). **(F)** Quantitative analysis of CD206^+^-α-SMA^+^ cells. **(G)** Monocytes of WT or Jmjd3-cKO mice after IL-4 (50 ng/ml) or IRF4 overexpression were stained for CD206 (red) and α-SMA (green). **(H)** Quantitative analysis of CD206^+^-α-SMA^+^ cells. **(I)** Western blots bands of protein expression of Jmjd3, IRF4, fibronectin, and α-SMA in cultured monocytes. **(J)** Quantitative analysis of Jmjd3, IRF4, fibronectin, and α-SMA protein levels. **(K)** Monocytes of WT mice after IL-4 or (and) GSKJ4 treatment were stained for CD206 (red) and α-SMA (green). **(L)** Quantitative analysis of CD206^+^-α-SMA^+^ cells. *P<0.05 or **P<0.01; n = 6; Bar = 50 μm. WT, wild-type; cKO, conditional knockout; KO, knockout; HPF, high-power field.

IL-4/STAT6 axis significantly promotes bone marrow-derived myofibroblasts accumulation which is originated bone marrow-derived monocytes through M2 macrophage polarization (16). Also, IL-4/STAT/Jmjd3 signaling and Jmjd3/IRF4 axis is imperative for M2 macrophage polarization (20, 21). Thus, we investigated whether IL-4 treatment has an impact on Jmjd3/IRF4 signaling in cultured myeloid monocytes of mice. We showed that IL-4 stimulation significantly up-regulated the expressions of Jmjd3, IRF4, ECM, and α-SMA. Of note, myeloid Jmjd3 deletion or IRF4 knockout abolished these proteins expression (**Figures 10A–D**). Furthermore, the CD206^+^-α-SMA^+^ dual positive cells was markedly elevated in monocytes exposed to IL-4, whereas myeloid Jmjd3 deletion resulted in a notable reduction of these cells. However, overexpression of IRF4 abrogated the effect of myeloid Jmjd3 deletion on M2MMT (**Figures 10E–H**). GSKJ4, a Jmjd3 inhibitor, markedly suppressed IL-4-induced the levels of Jmjd3, IRF4, ECM and α-SMA *in vitro*. We also showed that GSKJ4 treatment significantly lowered the number of CD206^+^-α-SMA^+^ cells in cultured monocytes stimulated by IL-4 (**Figures 10I–L**). Additionally, BMDMs were stimulated with TNF-α. However, it did not promote MMT (**Supplementary Figures S1D, E**). These data suggest that IL-4/Jmjd3/IRF4 axis regulates activation of myeloid fibroblast and differentiation of M2 macrophage to myofibroblast.

## Discussion

Bone marrow-derived myofibroblasts and M2-like phenotype macrophage contribute to kidney fibrogenesis (15, 16, 39, 40). Identifying the mechanisms that initiate myeloid fibroblasts activation and drive macrophages to pro-fibrotic M2 phenotype is a crucial step toward developing effective therapeutics. Recent data reveal that IL-4Rα/STAT6 signaling pathway drives bone marrow-derived fibroblasts and pro-fibrotic M2 macrophages accumulation in renal fibrosis (16). Jmjd3 has been identified as a direct downstream target of IL-4/STAT6 signaling pathway (20). In addition, IRF4 has been demonstrated to be a target gene of Jmjd3 (21). Nevertheless, the role of Jmjd3/IRF4 signaling in myeloid fibroblasts activation and M2MMT in fibrotic kidney remains unclear. This study is our continuous work. We demonstrate that Jmjd3 deficiency in myeloid cells or genetic ablation of IRF4 suppresses the accumulation of myeloid fibroblasts and impairs M2MMT in kidney fibrogenesis.

Jmjd3, a member of the Jumonj protein family, has been demonstrated to be a histone demethylase (19, 41). Accumulating data has shown that inhibition of Jmjd3 ameliorates fibrotic disorder. A study reveals that targeted inhibition of Jmjd3 suppresses activation of systemic sclerosis fibroblasts and protects mice against fibrosis induced by bleomycin or topoisomerase-I (29). In addition, inhibition of Jmjd3 impairs neonatal rat cardiac fibroblasts activation and cardiac fibrosis following myocardial infarction (33). Evidence shows that STAT6 directly bound to the Jmjd3 promoter region and Jmjd3 is a direct downstream target of STAT6 (20). Furthermore, Jmjd3 mediates the transition of bone marrow-derived monocytes to M2 macrophage phenotype, which depends on IL-4/STAT6 signaling (20). We and others have previously demonstrated that IL-4Rα/STAT6 signaling exerts an important impact on myeloid fibroblasts activation, macrophage M2 polarization, and kidney fibrosis progression (15, 16). To continue our research on this signaling pathway, we have investigated the role of Jmjd3 in renal fibrosis in the current study. Consistent with our previous results, we reveal that myeloid Jmjd3 deletion or pharmacological inhibition of Jmjd3 notably impairs the progression of kidney fibrosis in folic acid or obstructive nephropathy.

Myofibroblasts are α-SMA positive cells that produce ECM proteins, leading to collagen deposition and kidney fibrosis (42). Cumulative efforts to understand the origin of myofibroblasts in fibrotic kidney have consistently highlighted a crucial role of bone marrow-derived fibroblasts activation (40). Our findings show that myeloid Jmjd3 deletion or Jmjd3 inhibitor markedly reduces the accumulation of myeloid myofibroblasts, decreases the levels of fibronectin, and attenuates collagen deposition. In agreement with the data of animal models, myeloid Jmjd3 deletion or Jmjd3 inhibitor treatment abrogated the myofibroblast phenotype of bone marrow-derived monocytes stimulated by IL-4. These findings suggest that Jmjd3 mediates the activation of myeloid fibroblasts and kidney fibrosis development.

Macrophage M2 polarization has been proved involve in various fibrotic diseases (43–46). We have previously demonstrated that inhibition of IL-4Rα/STAT6 signaling impairs macrophages M2 polarization and attenuates renal fibrosis (16). A study has reported that Jmjd3 inhibition protects mice from pulmonary fibrosis by the regulation of pro-fibrotic M2 gene expression (31). Recently, M2 macrophages to myofibroblasts transformation has been proved, which profoundly contributes to the progression of renal fibrosis (11, 13). In this work, our data show myeloid Jmjd3 deletion or Jmjd3 inhibitor suppresses differentiation of M2 macrophages into myofibroblasts in renal fibrosis. Furthermore, Jmjd3 deletion or Jmjd3 inhibitor prevents the differentiation of M2 macrophages to myofibroblasts in cultured monocytes. It is well established that IL-4 or TGF-β can induce macrophage to myofibroblast transition (14, 37). However, we show that TNF-α did not trigger the transition of macrophage to myofibroblast. These results suggest that Jmjd3 in myeloid cells contributes to kidney fibrosis by the regulation of M2MMT.

As a hemopoietic-specific transcription factor, IRF4 is crucial for myeloid and lymphoid lineage progression (47). It is well established that IRF4 is a downstream target gene of Jmjd3 (21). The role of Jmjd3/IRF4 axis in many pathophysiological microenvironments has been explored intensively (21, 26, 28, 48). Recent data reveal that IRF4 has been implicated in fibrotic disorder. Sasaki et al. have reported that myeloid IRF4 deletion attenuates ischemia-induced renal fibrosis (38). Our recent work also reveals that IRF4 deficiency protects against inflammation and kidney fibrosis in folic acid nephropathy (24). To further understand the signaling pathway mechanism of Jmjd3 in kidney fibrosis, we investigate whether IRF4 mediates the anti-fibrotic effects of Jmjd3 inhibition in renal fibrosis. We show that folic acid or obstructive injury markedly induces expression of IRF4 in the kidneys, whereas myeloid Jmjd3 deletion or Jmjd3 inhibitor significantly reduces the levels of IRF4. Additionally, myeloid Jmjd3 deletion or GSKJ4 treatment decreases expressions of IRF4, fibronectin, and α-SMA in IL-4-stimulated monocytes. Moreover, IRF4 deficiency inhibits collagen deposition and myeloid myofibroblasts accumulation in obstructive nephropathy. These data indicate Jmjd3/IRF4 axis is highly activated and has an important impact on myeloid fibroblasts activation in progressive kidney fibrosis.

In terms of IRF4 has a critical role of regulating immune cells function and initiating macrophage M2 polarization (21, 49, 50). We next investigate whether IRF4 affect myeloid myofibroblast accumulation and M2MMT in kidneys with obstructive injury. Our data reveal that IRF4 ablation protects against the activation of bone marrow-derived fibroblasts and impedes M2MMT in obstructive kidneys. To strengthen the notion of bone marrow-derived cells exerts a significant impact on kidney fibrosis, we performed bone marrow transplantation experiment. The results demonstrated that IRF4 in myeloid cells triggers myeloid fibroblasts differentiation and kidney progression after obstructive injury.

A study showed that inhibition of Jmjd3 exacerbated renal fibrosis (34). However, the investigators used a murine model of 5/6 surgical nephrectomy in that study, which is different from the murine model of folic acid nephropathy. Furthermore, Jmjd3^flox^/^flox^ Foxd1Cre^+^ mice were used in their study, whereas Jmjd3^flox^/^flox^Lyz2Cre^+^ mice were used in our study. Expression of Jmjd3 in different cell types might display a variety of functional phenotypes in kidney fibrosis following stress.

In this study, we have not detected that on which myeloid cells mainly express Jmjd3 or IRF4 in injured kidneys. Whether myeloid Jmjd3 deletion has an impact on dendritic cell or neutrophil activation requires further investigation. This is a limitation of this study.

**Figure 11:**
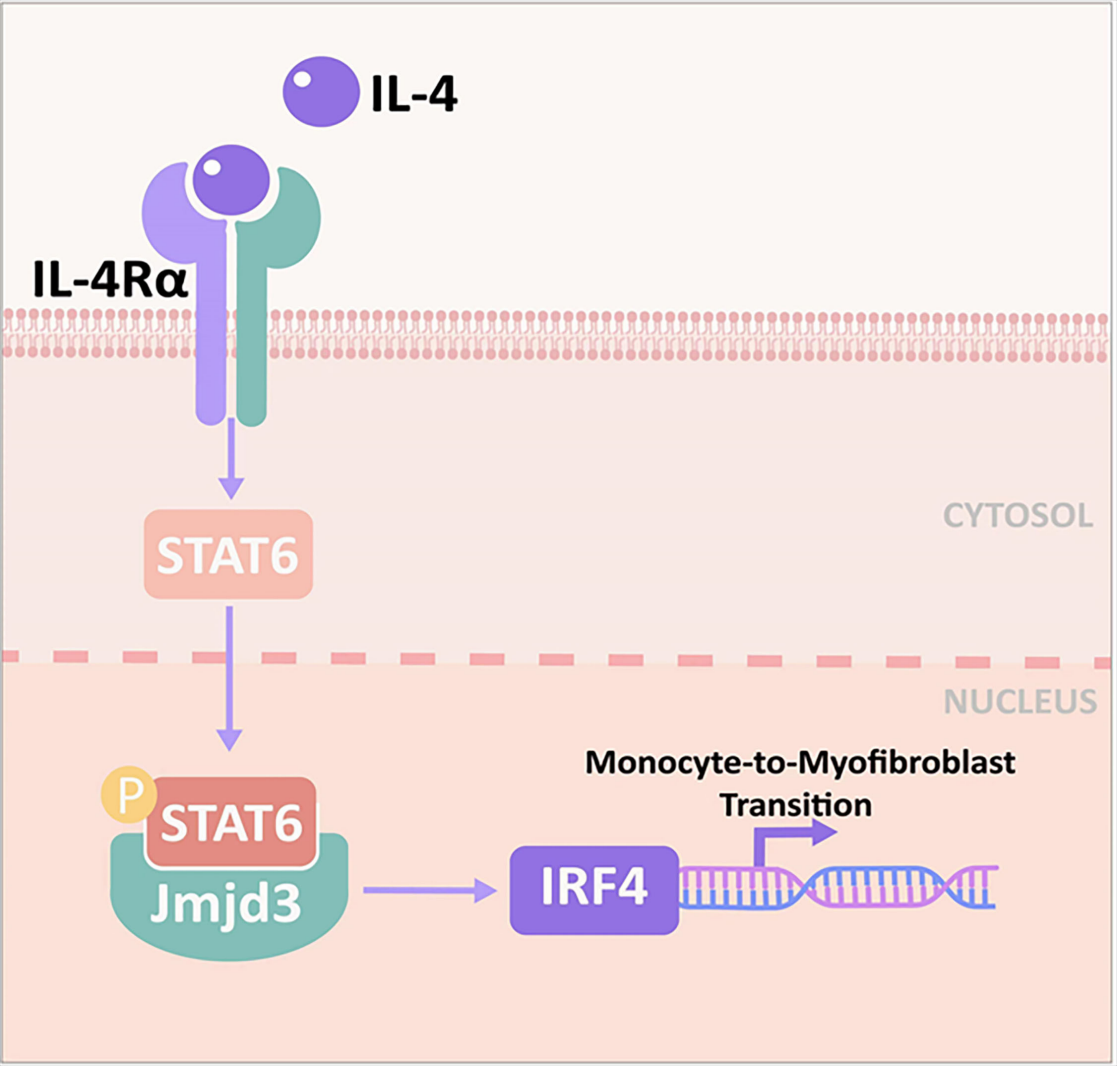
Proposed model for the role of Jmjd3/IRF4 axis in IL-4–induced myeloid activation and kidney fibrogenesis. IL-4 stimulates Jmjd3/IRF4 signaling, leading to monocytes to myofibroblasts transition and kidney fibrosis.

Taken together, we demonstrate that Jmjd3/IRF4 axis plays a crucial role in bone marrow fibroblasts differentiation and renal fibrosis progression. Following stress, Th2 cytokines such as IL-4 are released, IL-4 stimulates Jmjd3/IRF4 axis, leading to myeloid fibroblasts activation and M2MMT, and kidney fibrogenesis (**Figure 11**). Targeting Jmjd3/IRF4 signaling may be a promising therapeutic strategy in renal fibrosis.

## Data availability statement

The original contributions presented in the study are included in the article/**Supplementary Material**. Further inquiries can be directed to the corresponding authors.

## Ethics statement

The animal study was reviewed and approved by Animal Ethics Committee of Sun Yat-sen University (No. 2017-692).

## Author contributions

HL and XS conceived and designed research. BL, WY, JN, YG, SF and HL performed experiments. WY and SW analyzed data. HL and BL drafted manuscript. WY and XS edited and revised manuscript. All authors approved final version of manuscript.

## Acknowledgments

We thank Chaoqun Zhong for her assistance with the schematic diagram. This study was supported by grants from National Natural Science Foundation of China (No. 81871539), Natural Science Foundation of Guangdong Province (No. 2021A1515011481), and Medical Scientific Research Foundation of Guangdong Province (No. A2020608).

## Conflict of interest

The authors declare that the research was conducted in the absence of any commercial or financial relationships that could be construed as a potential conflict of interest.

## Publisher’s note

All claims expressed in this article are solely those of the authors and do not necessarily represent those of their affiliated organizations, or those of the publisher, the editors and the reviewers. Any product that may be evaluated in this article, or claim that may be made by its manufacturer, is not guaranteed or endorsed by the publisher.

## Abbreviations

Jmjd3, Jumonji domain containing 3; IRF4, interferon regulatory factor 4; M2MMT, M2 macrophages to myofibroblasts transition; CKD, Chronic kidney disease; ECM, Extracellular matrix; ESRD, End-stage renal disease; JAK3, Janus kinase 3; STAT6, Signal transducer and activator of transcription 6; IL-4Rα, IL-4 receptor α; FA, folic acid; UUO, unilateral ureteral obstruction.
