# Supplementary material for: Jmjd3/IRF4 axis aggravates myeloid fibroblast activation and m2 macrophage to myofibroblast transition in renal fibrosis
Source: Front Immunol. 2022 Sep 8;13:978262. doi: 10.3389/fimmu.2022.978262 (PMC9494509; doi:10.3389/fimmu.2022.978262)
Supplement: Supplementary file 1 [file DataSheet_1.zip › supplementary materials/WB-RAW-DATA-Fron-Immuno1.PPTX]

## Slide 1
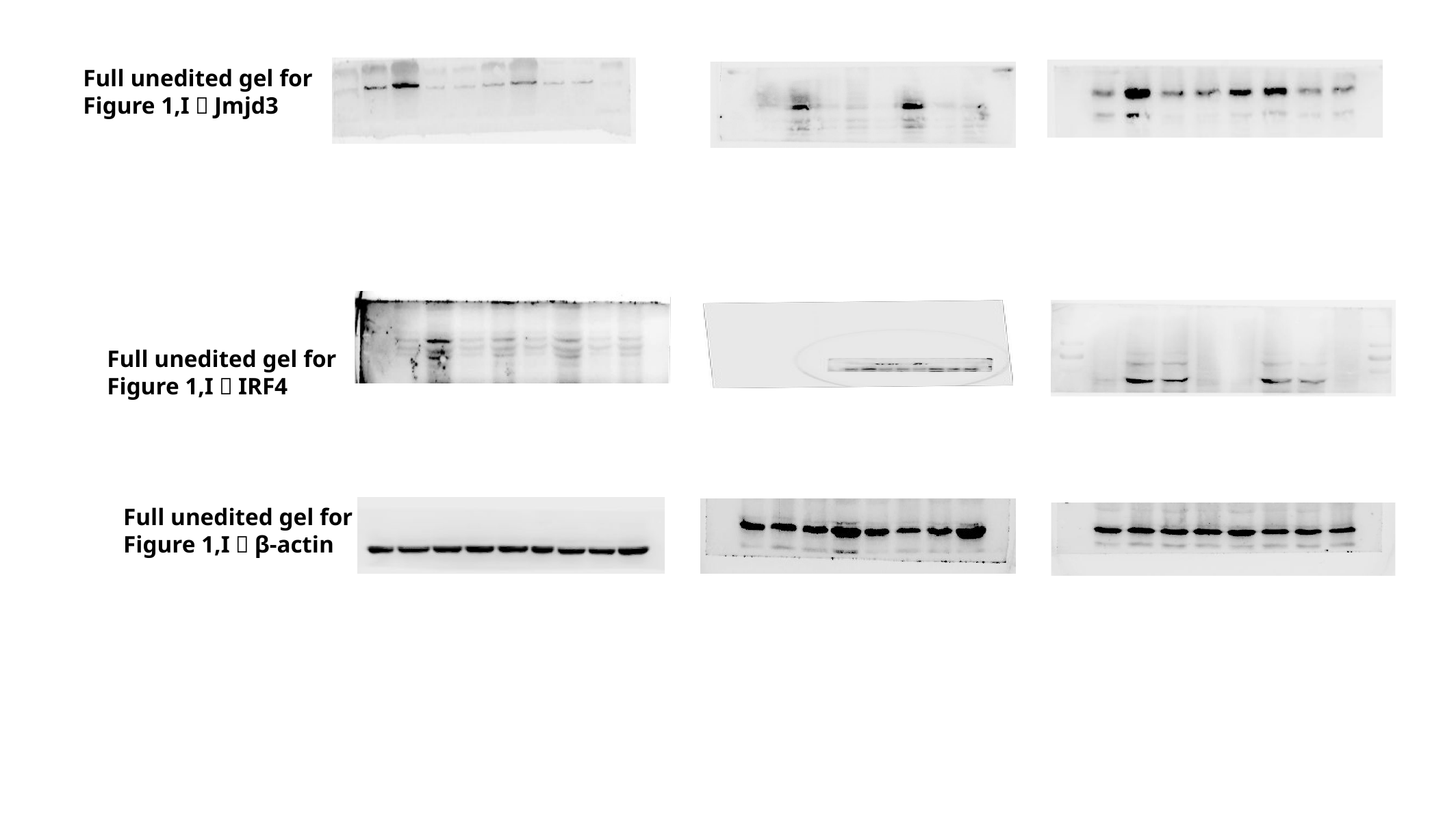

Full unedited gel for Figure 1,I：Jmjd3
Full unedited gel for Figure 1,I：IRF4
Full unedited gel for Figure 1,I：β-actin

## Slide 2
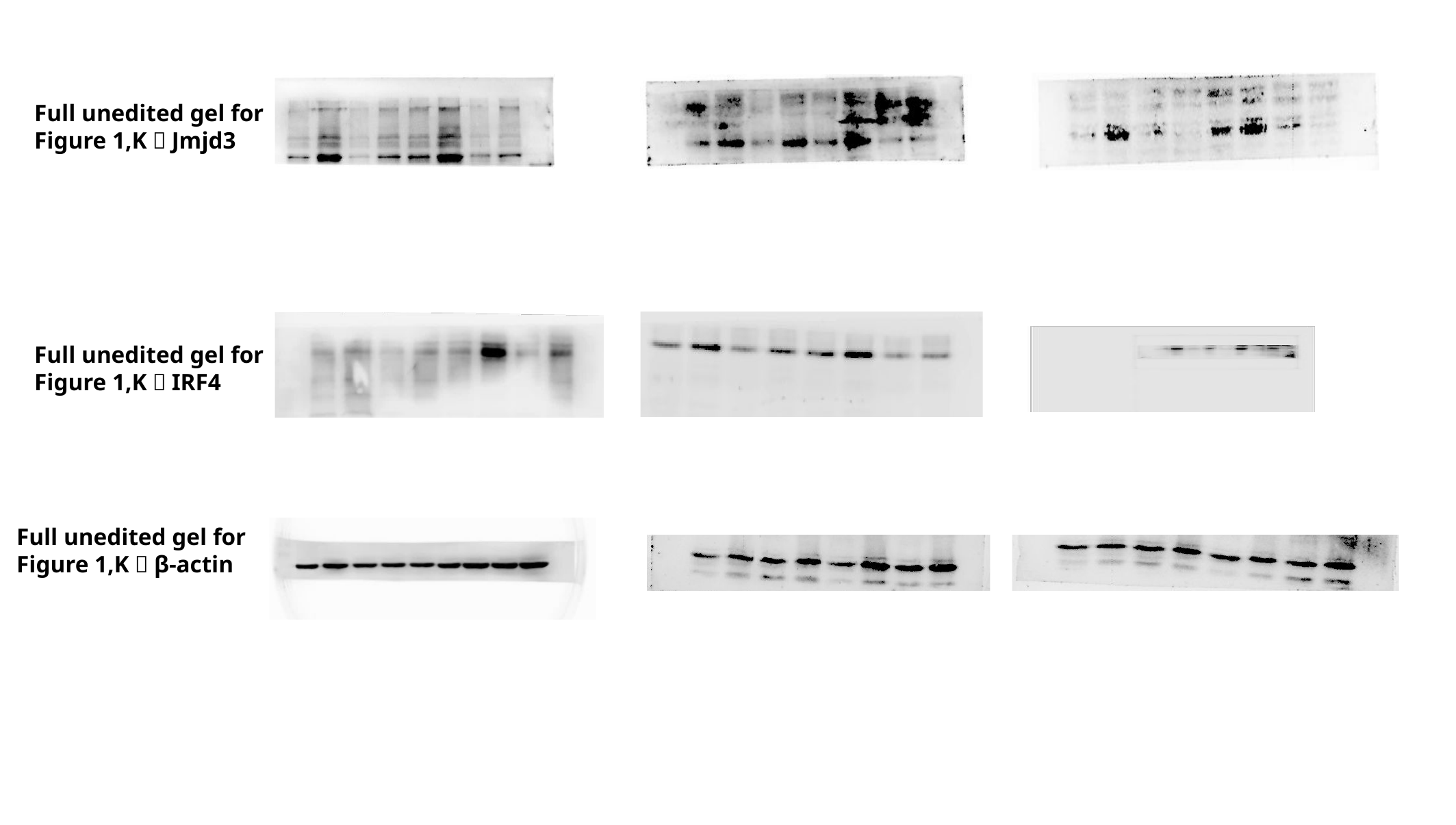

Full unedited gel for Figure 1,K：Jmjd3
Full unedited gel for Figure 1,K：IRF4
Full unedited gel for Figure 1,K：β-actin

## Slide 3
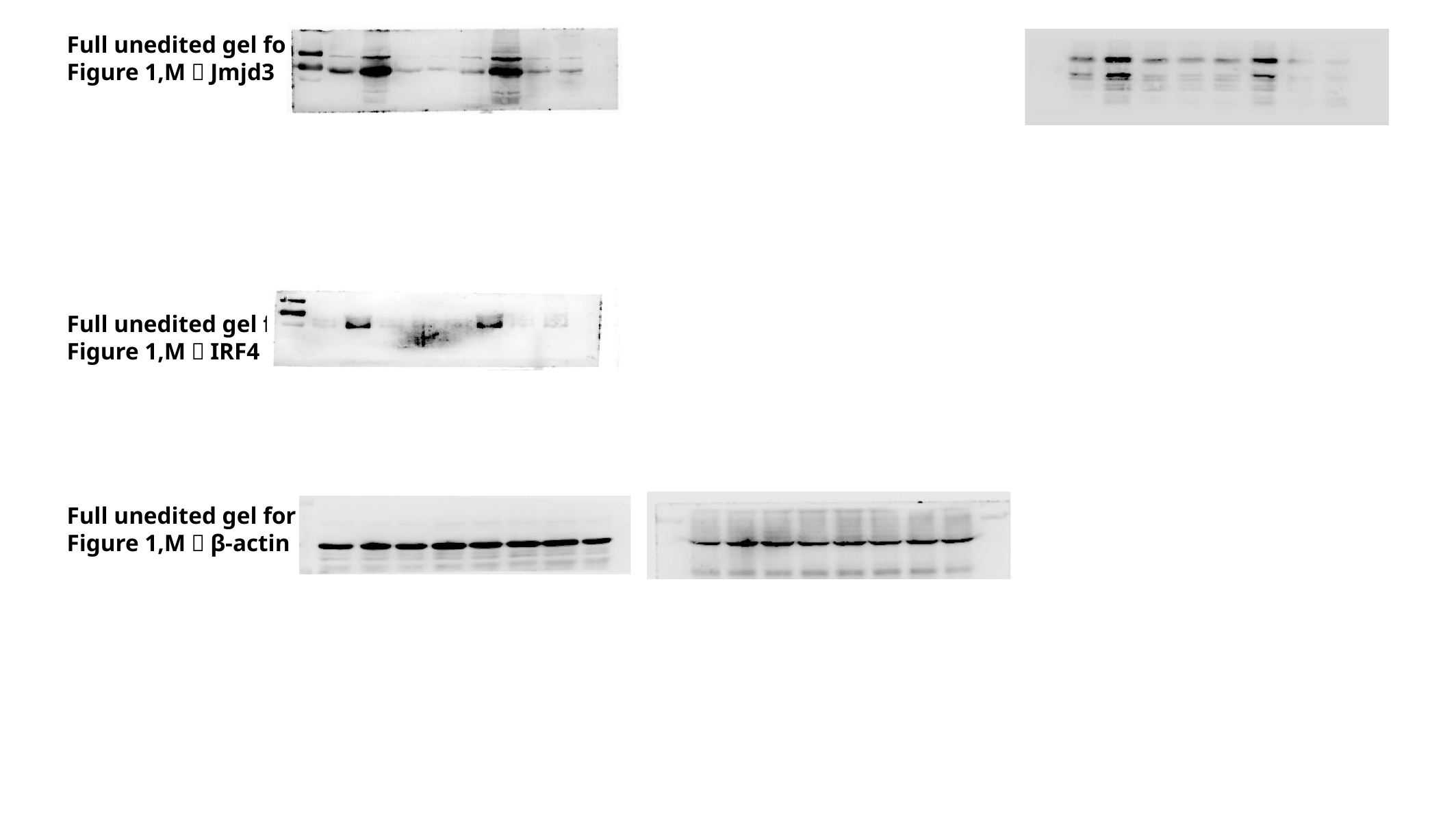

Full unedited gel for Figure 1,M：Jmjd3
Full unedited gel for Figure 1,M：IRF4
Full unedited gel for Figure 1,M：β-actin

## Slide 4
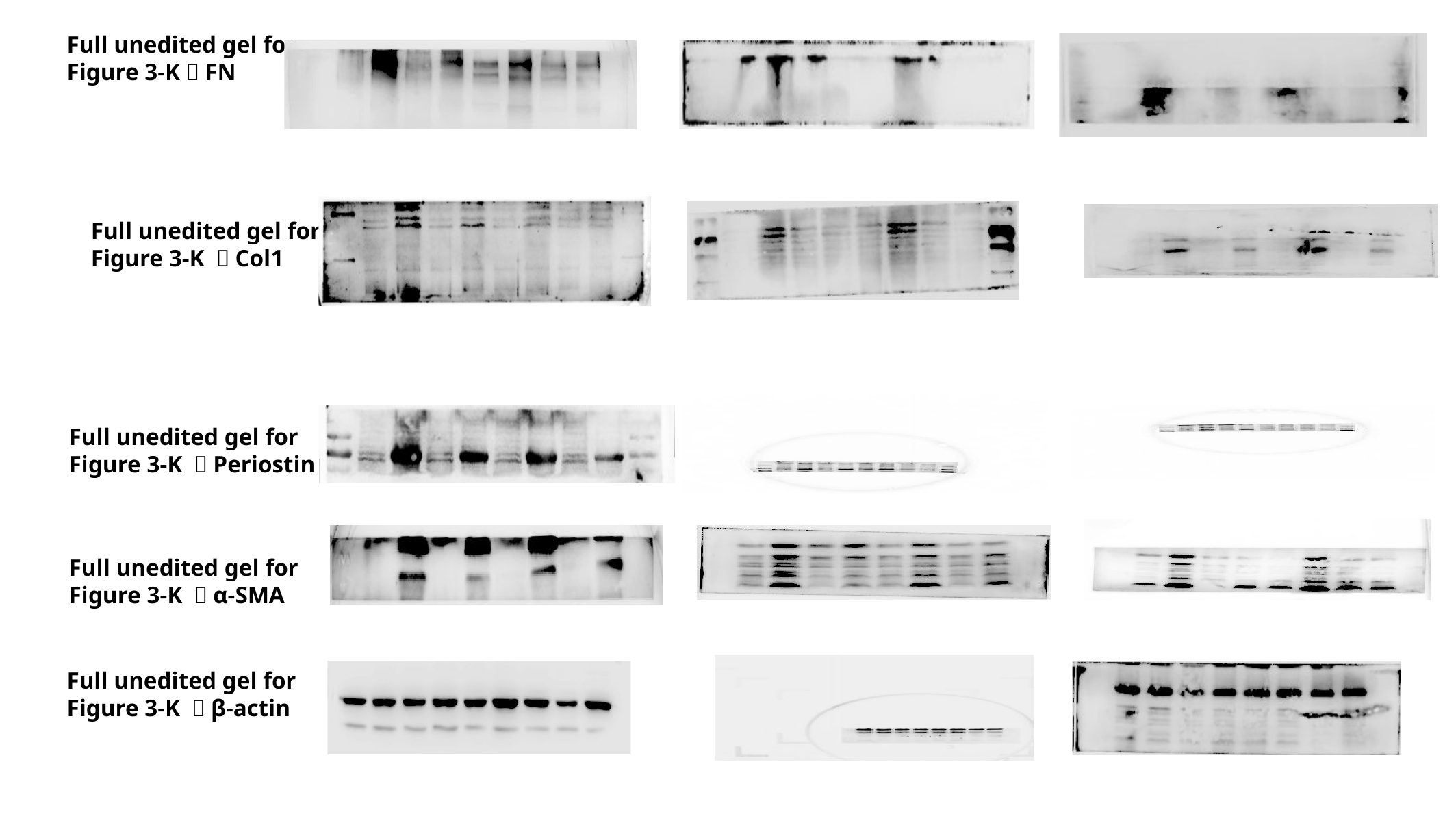

Full unedited gel for Figure 3-K：FN
Full unedited gel for Figure 3-K ：Col1
Full unedited gel for Figure 3-K ：Periostin
Full unedited gel for Figure 3-K ：α-SMA
Full unedited gel for Figure 3-K ：β-actin

## Slide 5
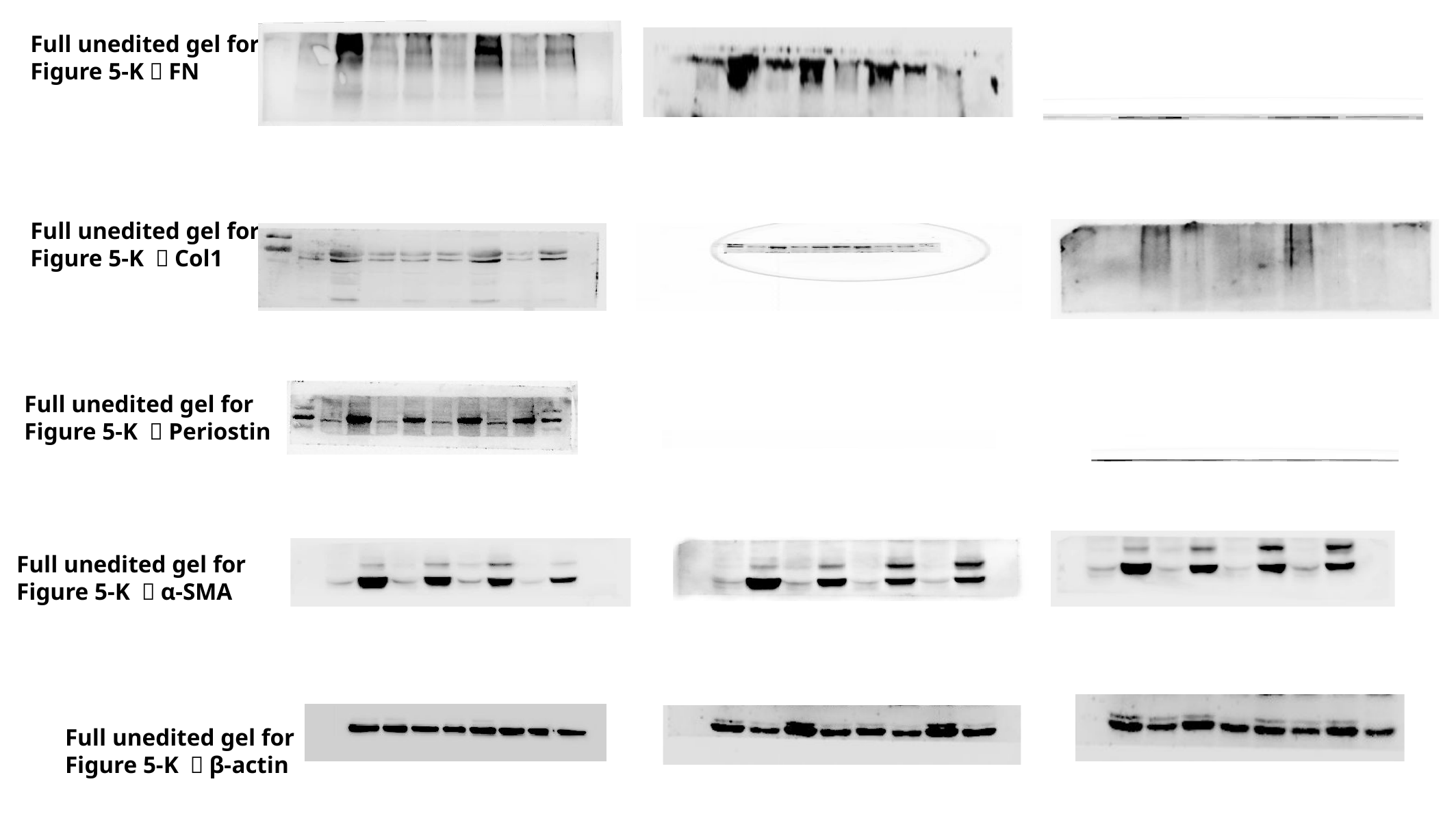

Full unedited gel for Figure 5-K：FN
Full unedited gel for Figure 5-K ：Col1
Full unedited gel for Figure 5-K ：Periostin
Full unedited gel for Figure 5-K ：α-SMA
Full unedited gel for Figure 5-K ：β-actin

## Slide 6
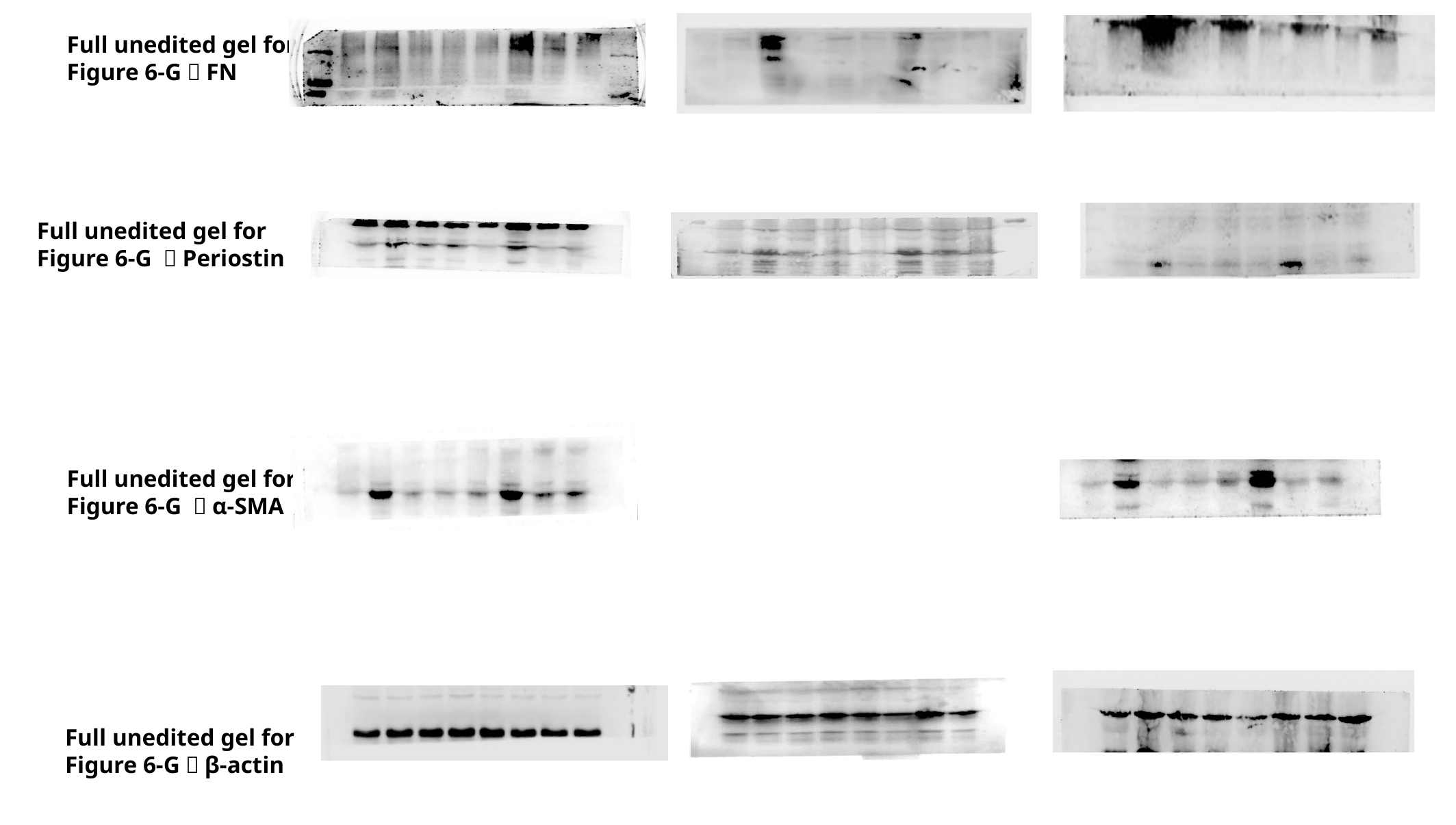

Full unedited gel for Figure 6-G：FN
Full unedited gel for Figure 6-G ：Periostin
Full unedited gel for Figure 6-G ：α-SMA
Full unedited gel for Figure 6-G：β-actin

## Slide 7
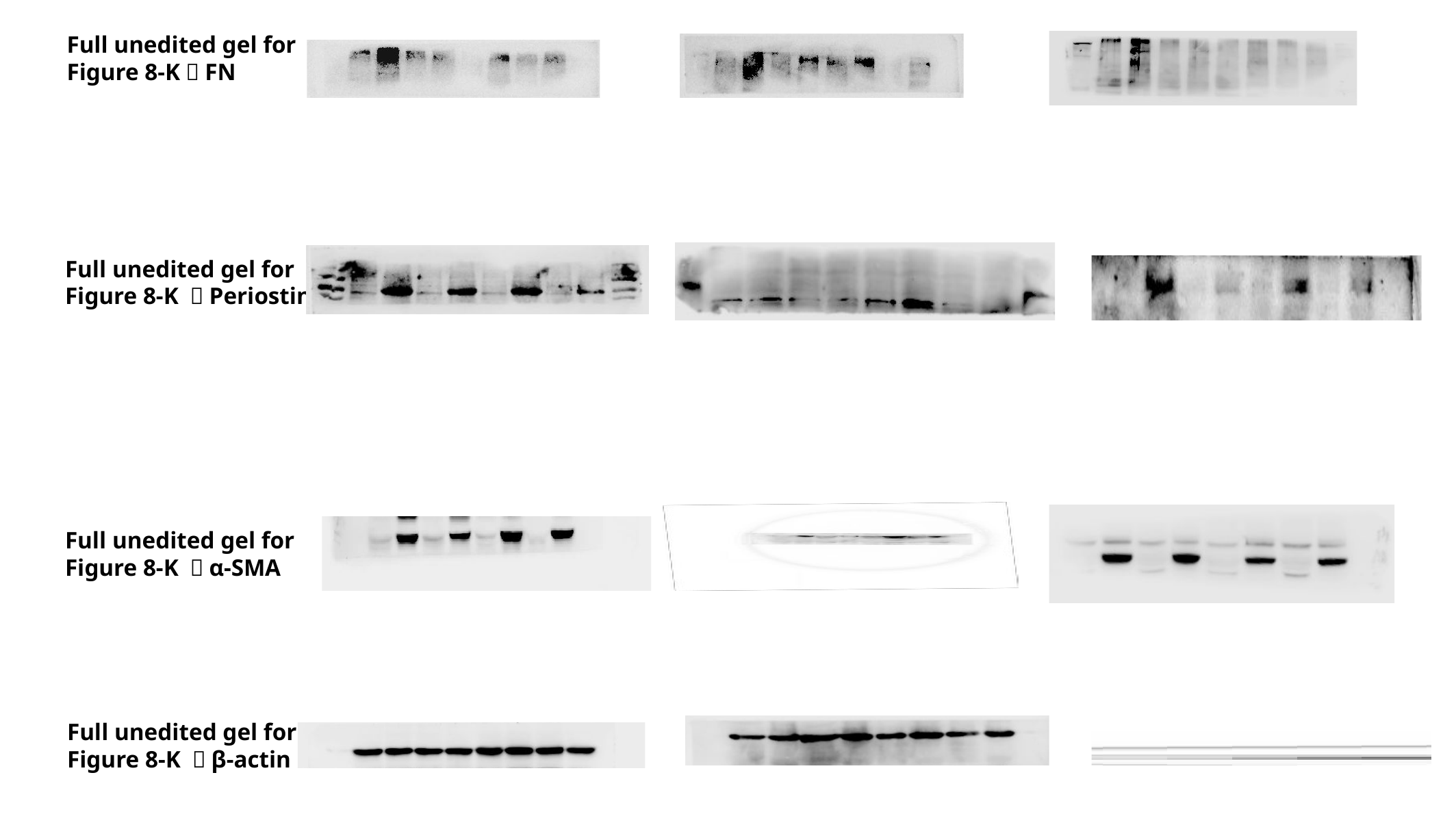

Full unedited gel for Figure 8-K：FN
Full unedited gel for Figure 8-K ：Periostin
Full unedited gel for Figure 8-K ：α-SMA
Full unedited gel for Figure 8-K ：β-actin

## Slide 8
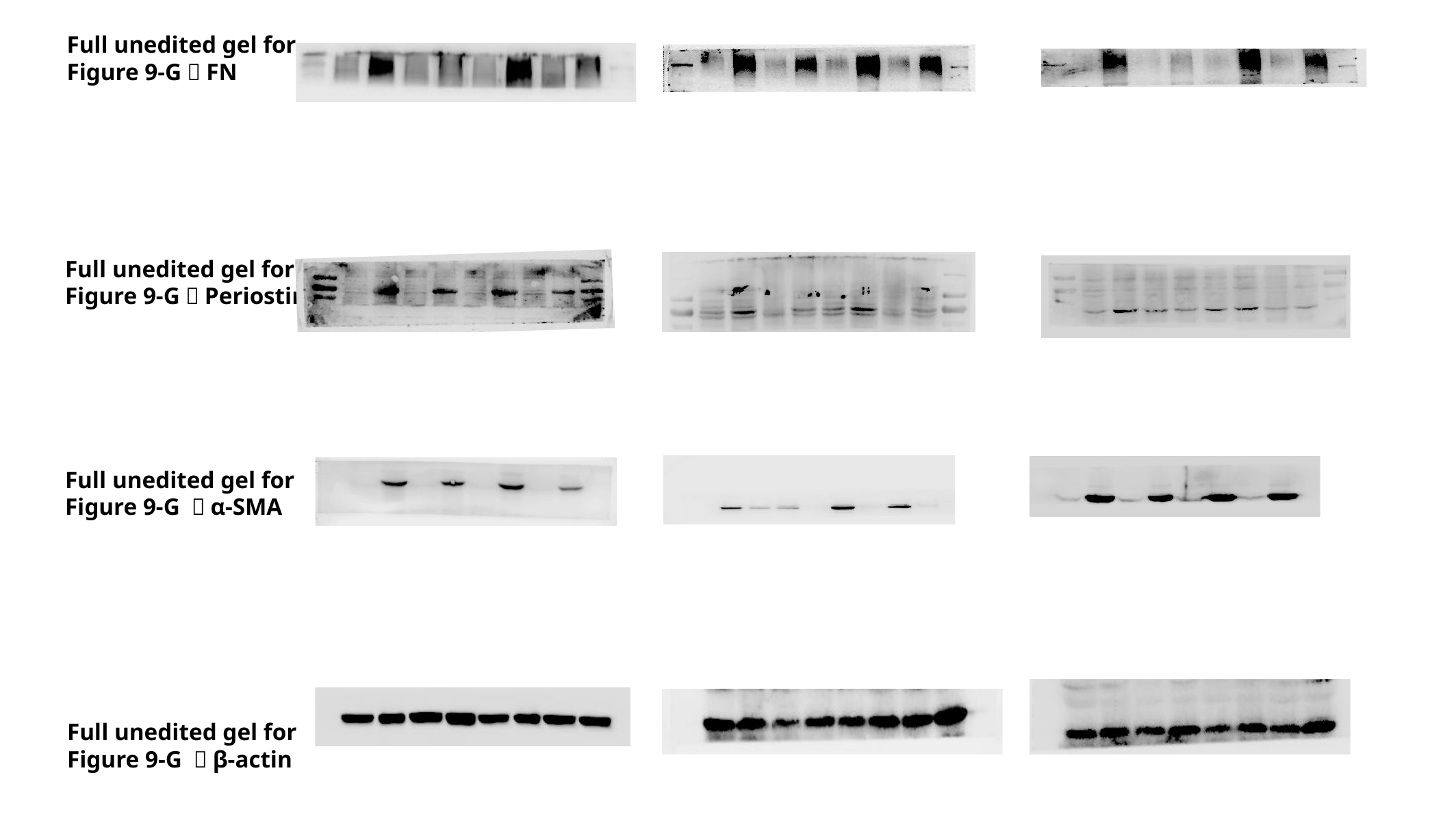

Full unedited gel for Figure 9-G：FN
Full unedited gel for Figure 9-G：Periostin
Full unedited gel for Figure 9-G ：α-SMA
Full unedited gel for Figure 9-G ：β-actin

## Slide 9
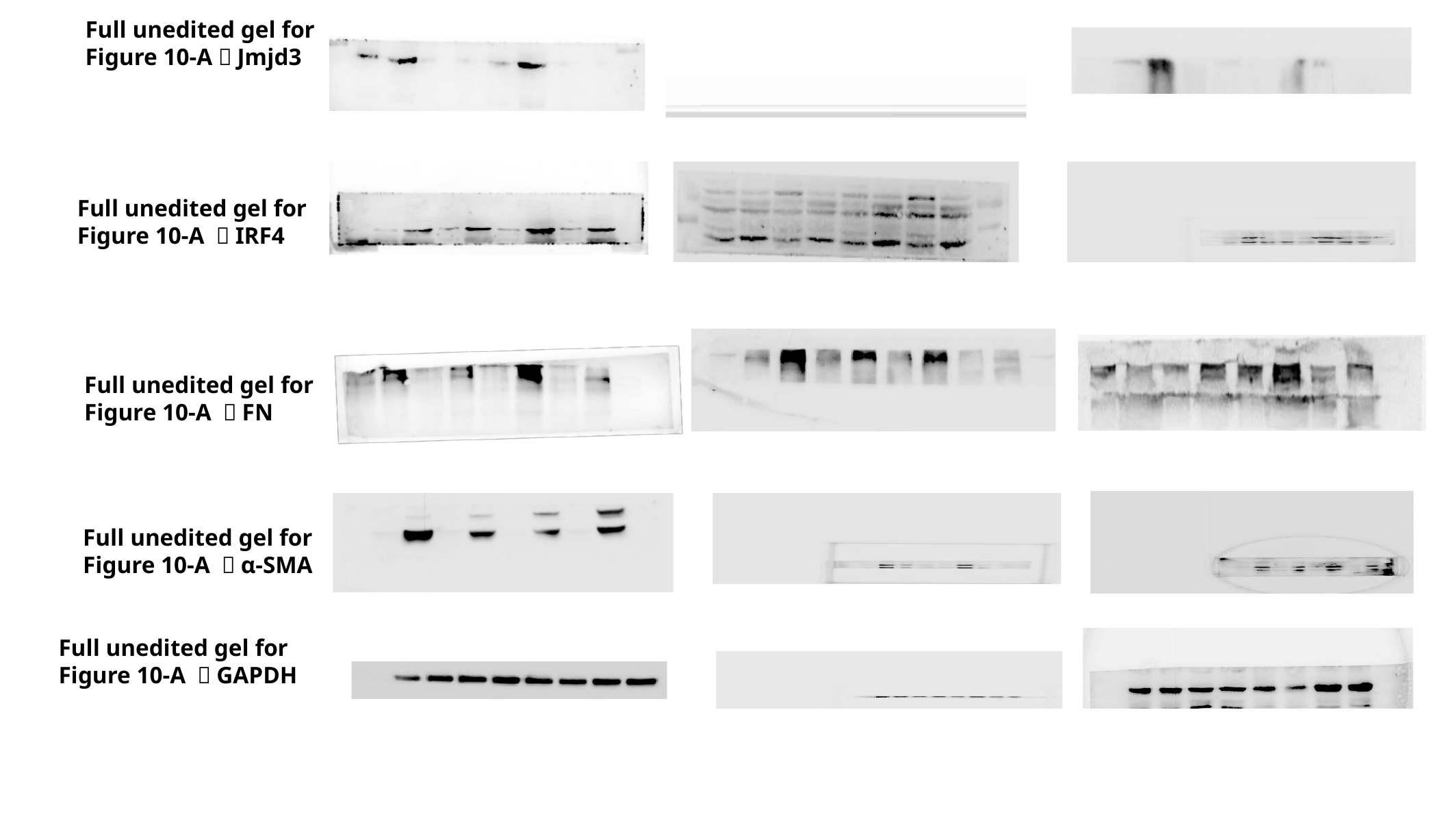

Full unedited gel for Figure 10-A：Jmjd3
Full unedited gel for Figure 10-A ：IRF4
Full unedited gel for Figure 10-A ：FN
Full unedited gel for Figure 10-A ：α-SMA
Full unedited gel for Figure 10-A ：GAPDH

## Slide 10
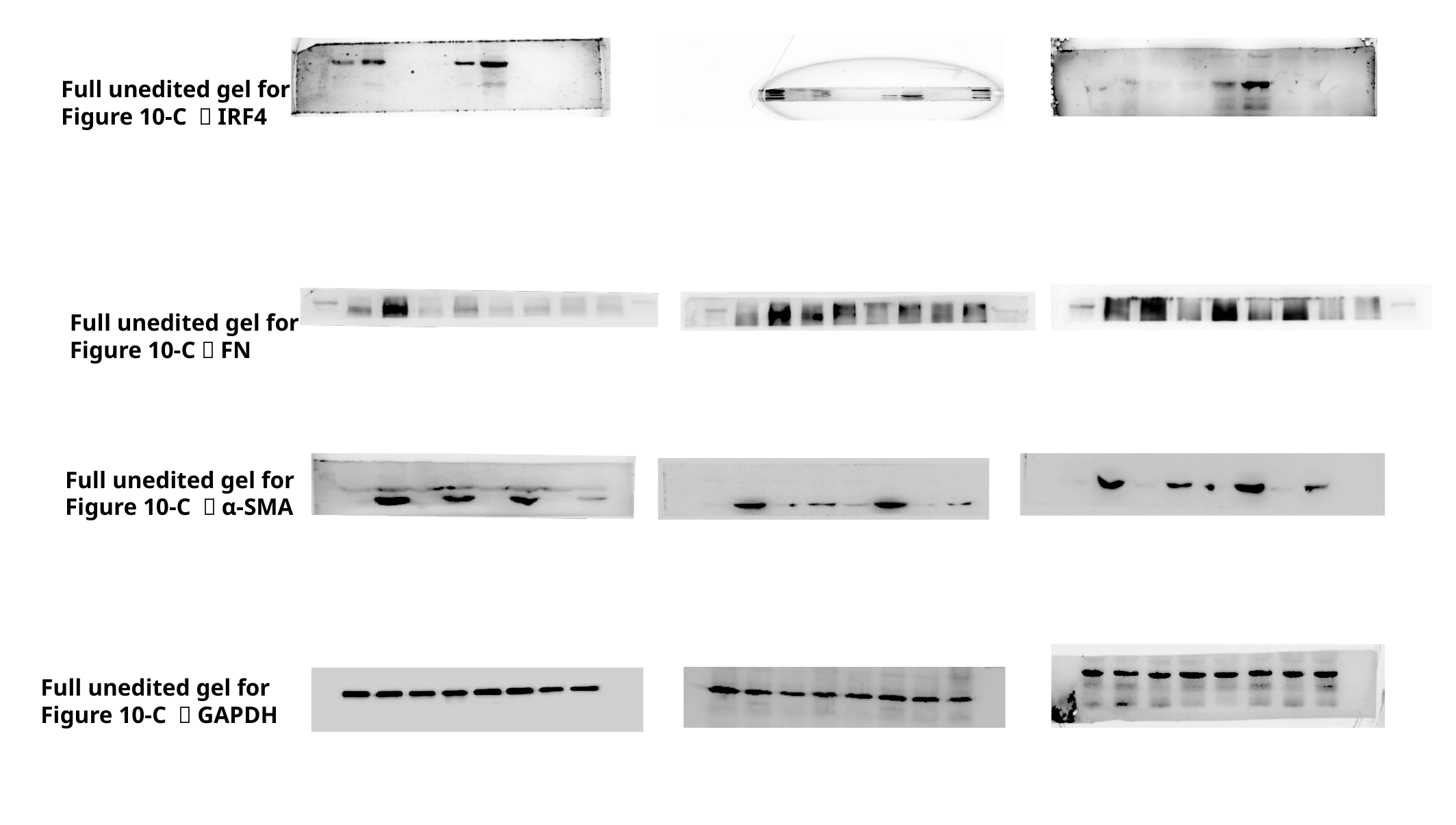

Full unedited gel for Figure 10-C ：IRF4
Full unedited gel for Figure 10-C：FN
Full unedited gel for Figure 10-C ：α-SMA
Full unedited gel for Figure 10-C ：GAPDH

## Slide 11
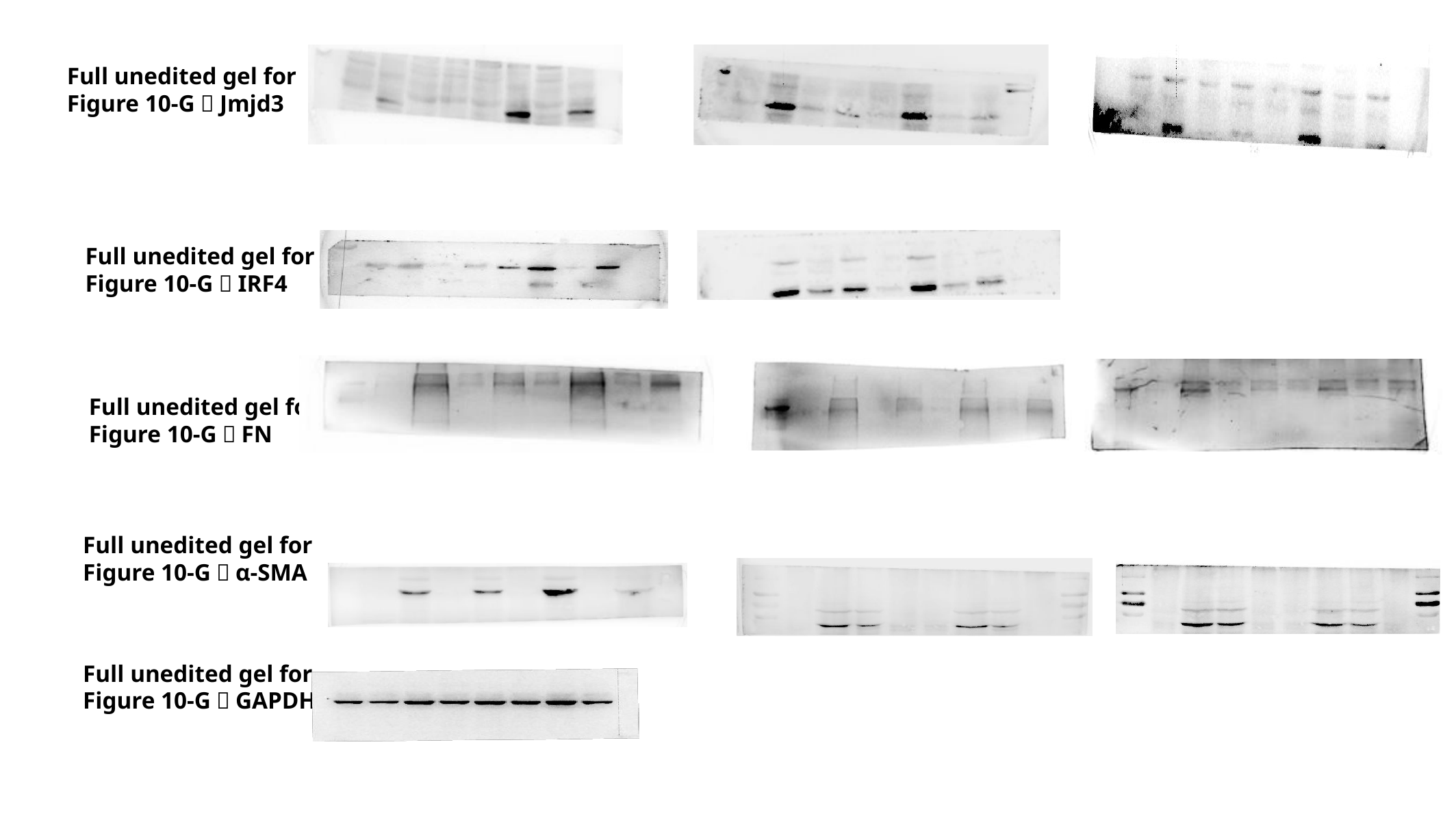

Full unedited gel for Figure 10-G：Jmjd3
Full unedited gel for Figure 10-G：IRF4
Full unedited gel for Figure 10-G：FN
Full unedited gel for Figure 10-G：α-SMA
Full unedited gel for Figure 10-G：GAPDH
