# Supplementary material for: Jmjd3/IRF4 axis aggravates myeloid fibroblast activation and m2 macrophage to myofibroblast transition in renal fibrosis
Source: Front Immunol. 2022 Sep 8;13:978262. doi: 10.3389/fimmu.2022.978262 (PMC9494509; doi:10.3389/fimmu.2022.978262)
Supplement: Supplementary file 1 [file DataSheet_1.zip › supplementary materials/supplementary figure/supplementary figure 1.pptx]

## Slide 1
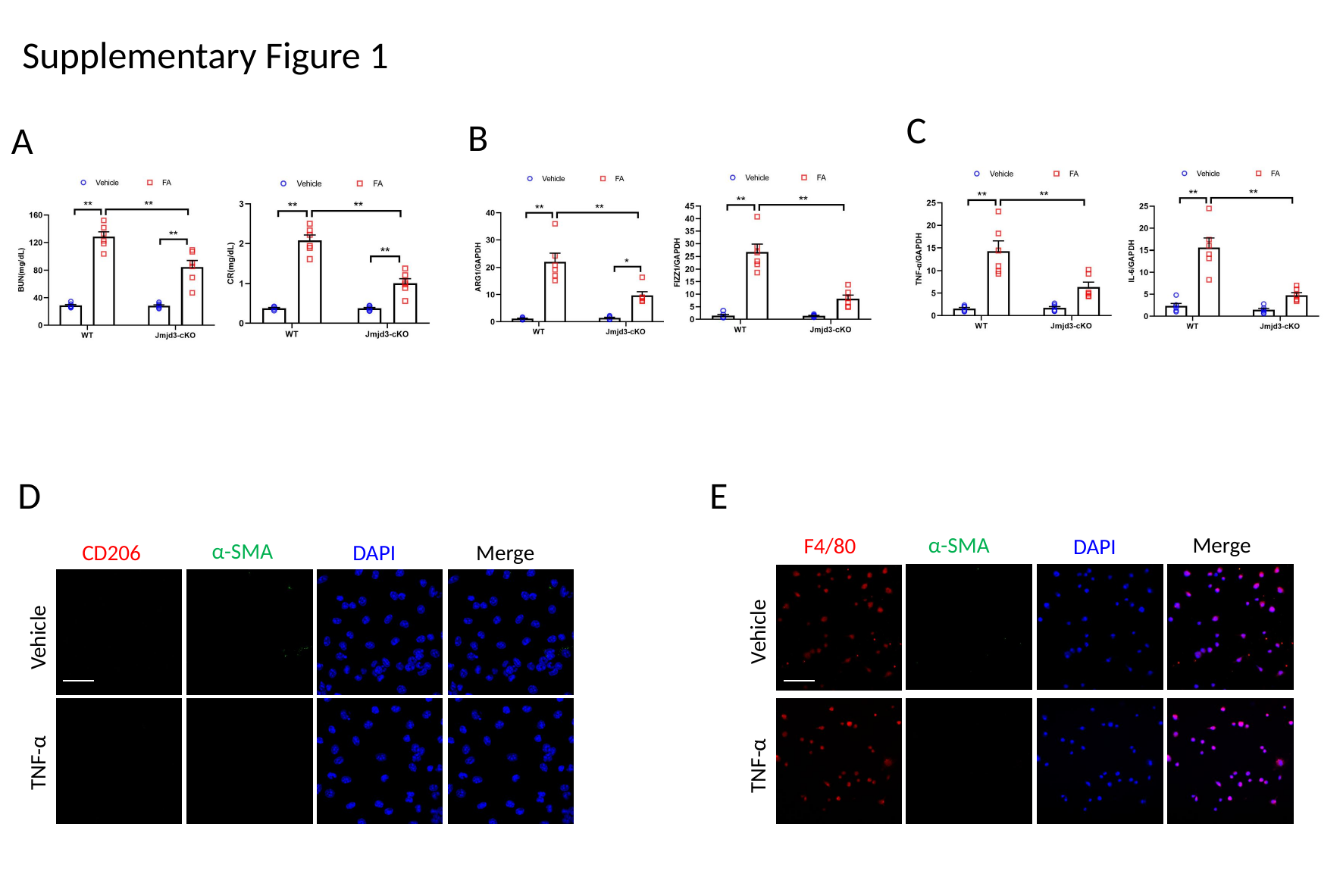

Supplementary Figure 1
C
B
A
E
D
Merge
α-SMA
F4/80
DAPI
α-SMA
CD206
DAPI
Merge
Vehicle
Vehicle
TNF-α
TNF-α
