# Supplementary figures and images for: Jmjd3/IRF4 axis aggravates myeloid fibroblast activation and m2 macrophage to myofibroblast transition in renal fibrosis
Source: Front Immunol. 2022 Sep 8;13:978262. doi: 10.3389/fimmu.2022.978262 (PMC9494509; doi:10.3389/fimmu.2022.978262)

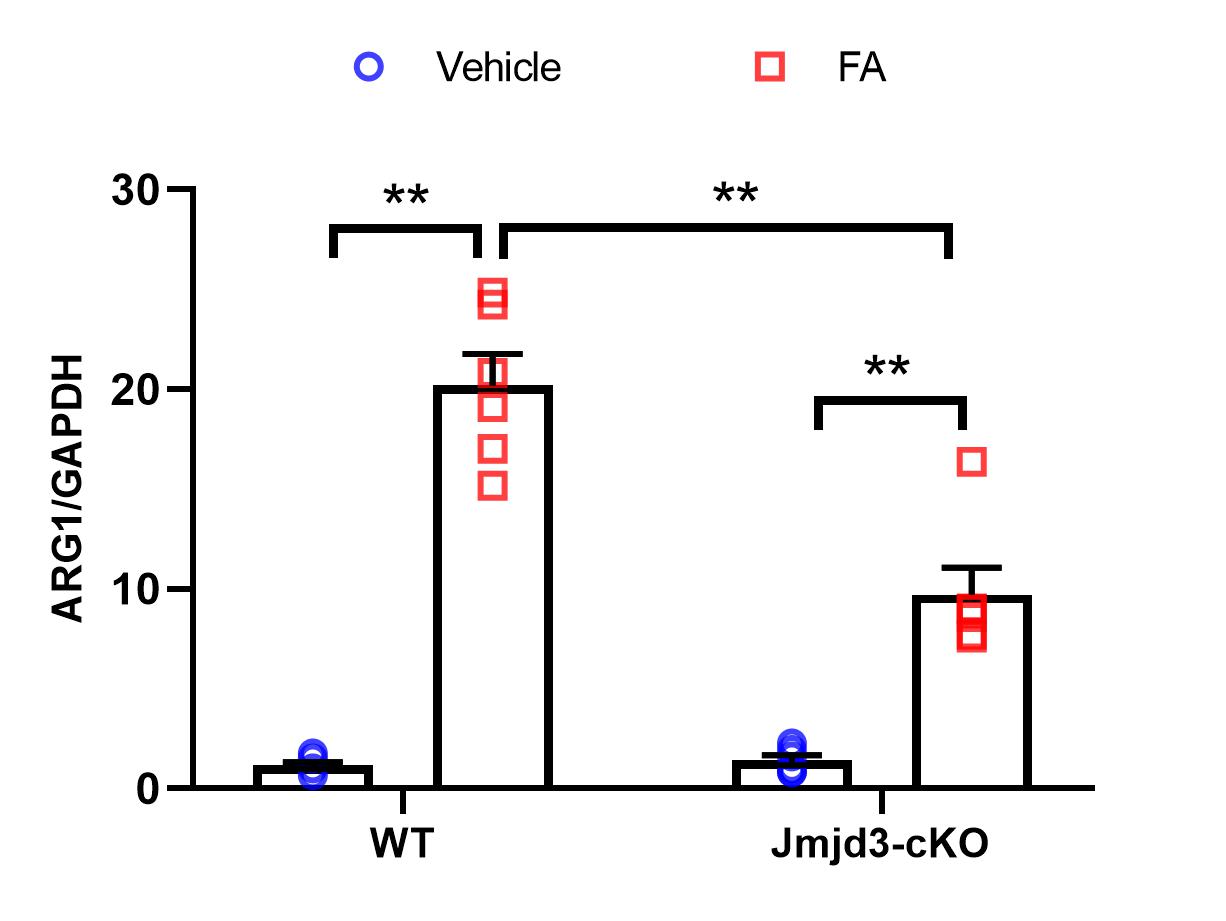

Supplement: Supplementary file 1 [file DataSheet_1.zip › supplementary materials/ARG1+FIZZ1/ARG1.jpg]

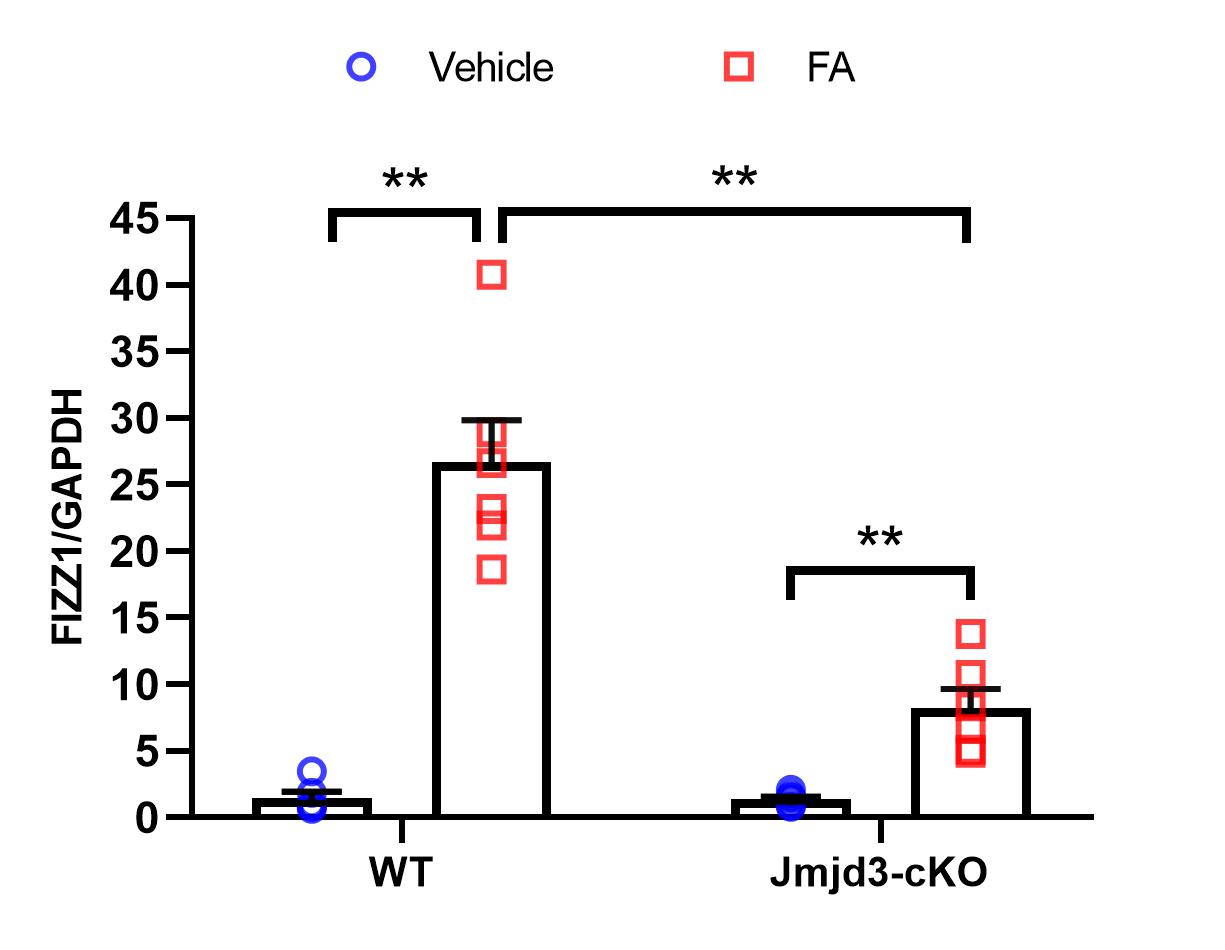

Supplement: Supplementary file 1 [file DataSheet_1.zip › supplementary materials/ARG1+FIZZ1/FIZZ1.jpg]

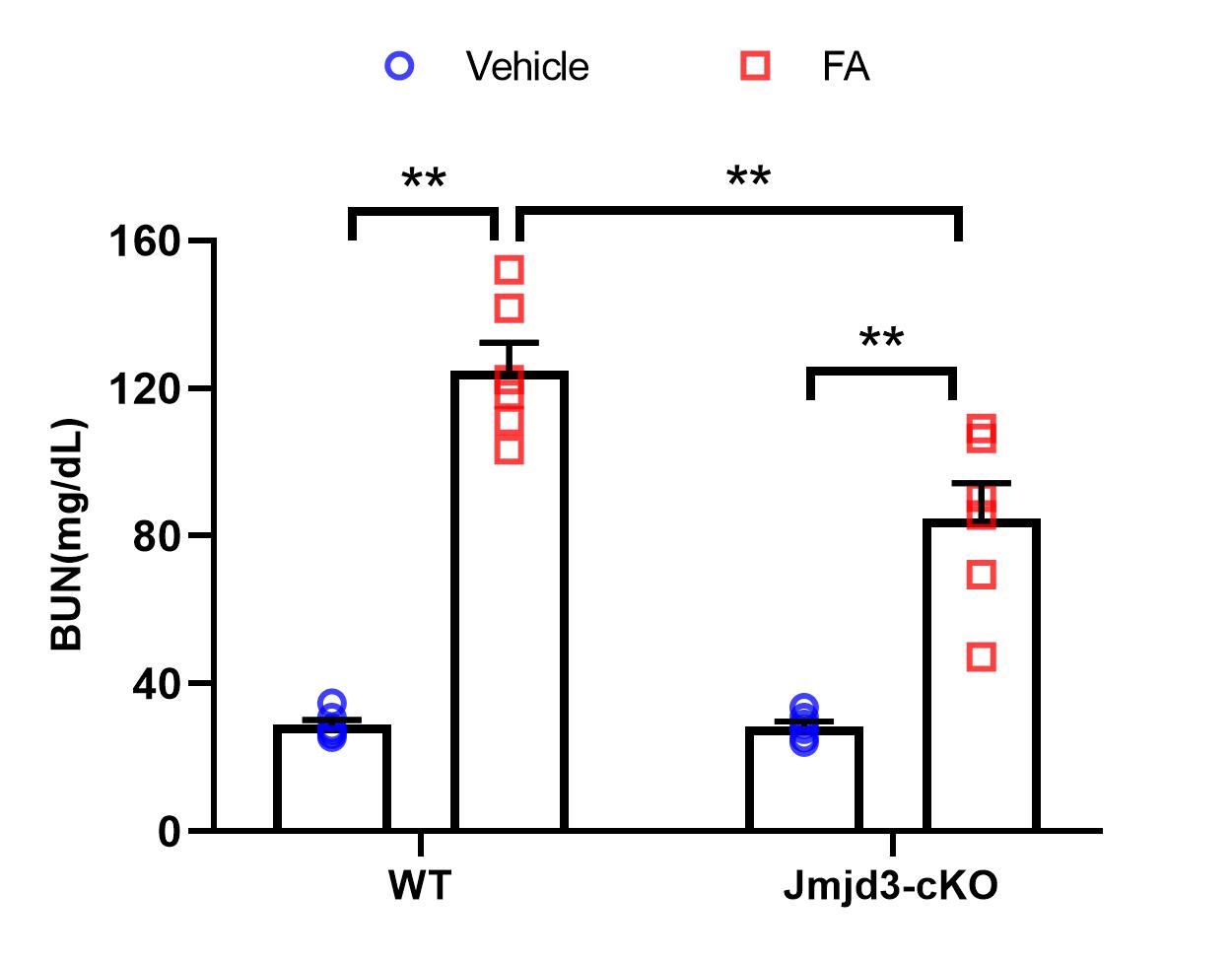

Supplement: Supplementary file 1 [file DataSheet_1.zip › supplementary materials/BUN+CR/BUN.jpg]

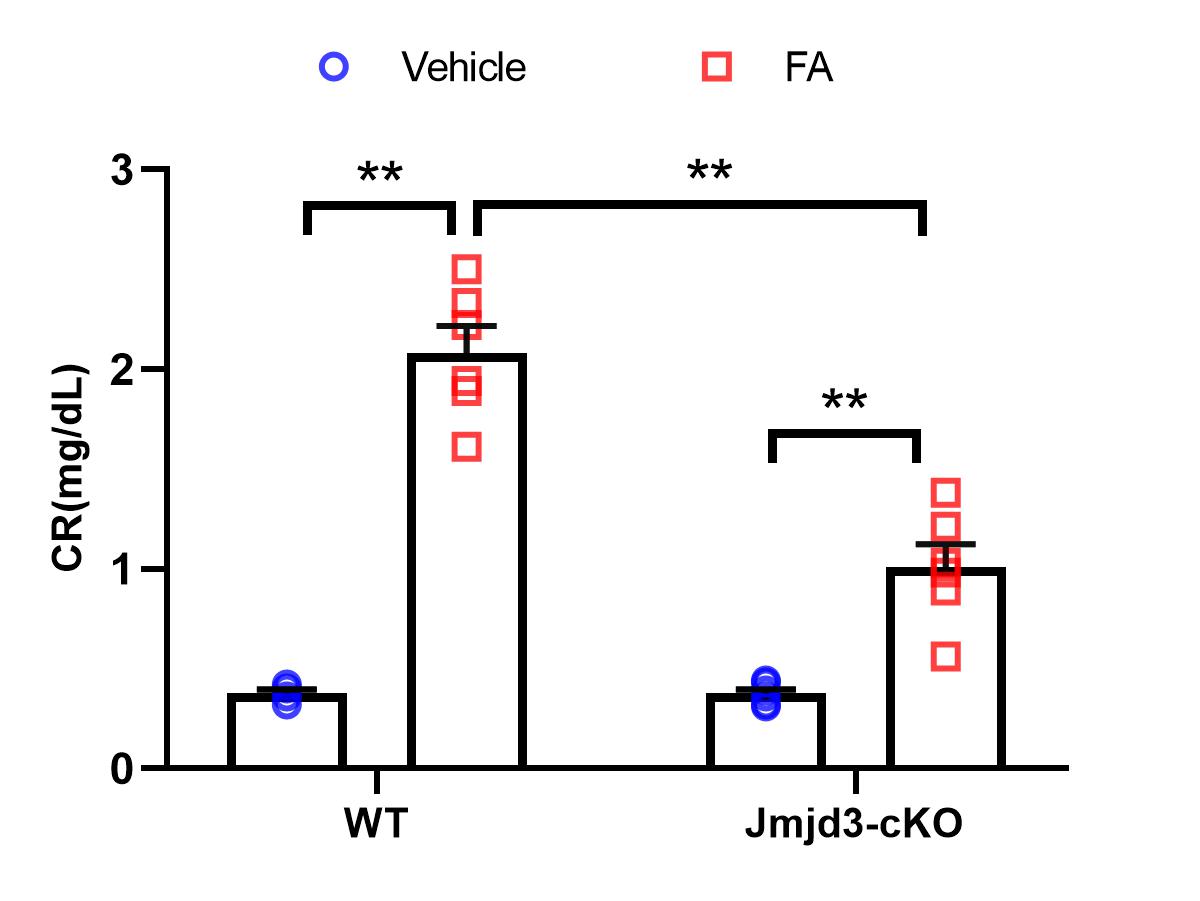

Supplement: Supplementary file 1 [file DataSheet_1.zip › supplementary materials/BUN+CR/CR.jpg]

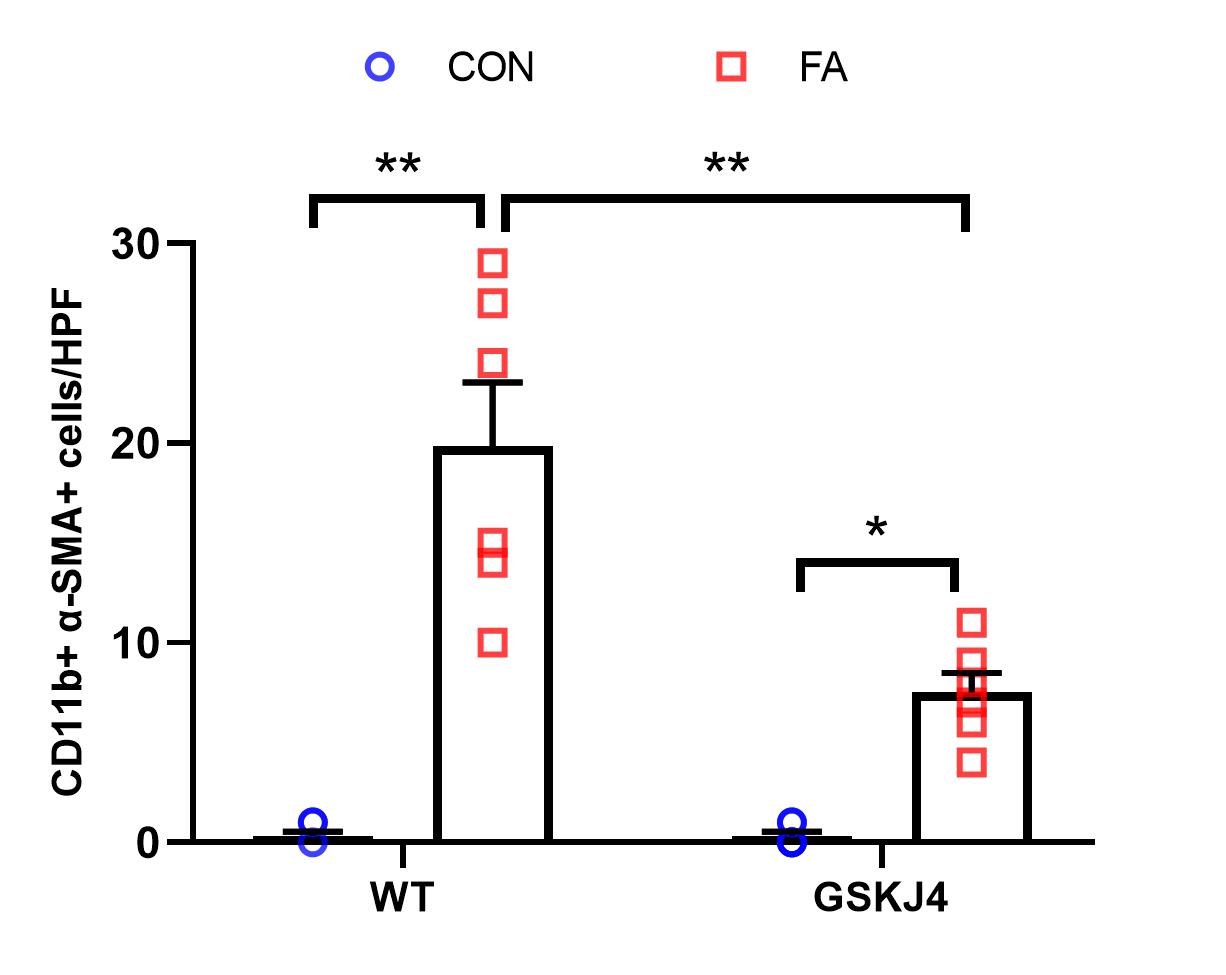

Supplement: Supplementary file 1 [file DataSheet_1.zip › supplementary materials/IF/1-CD11b-SMA-GSKJ4-FA.jpg]

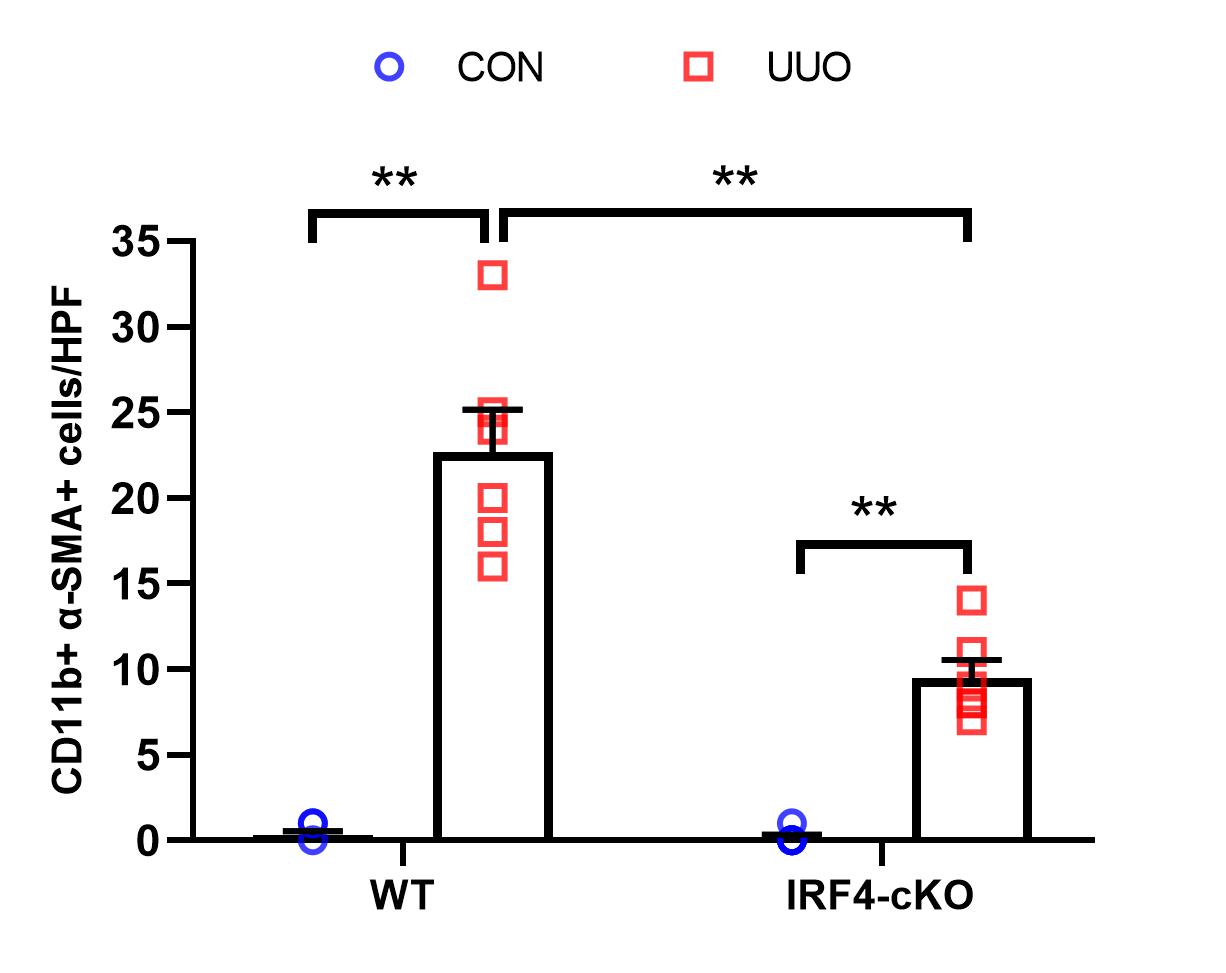

Supplement: Supplementary file 1 [file DataSheet_1.zip › supplementary materials/IF/1-CD11b-SMA-IRF4KO-UUO.jpg]

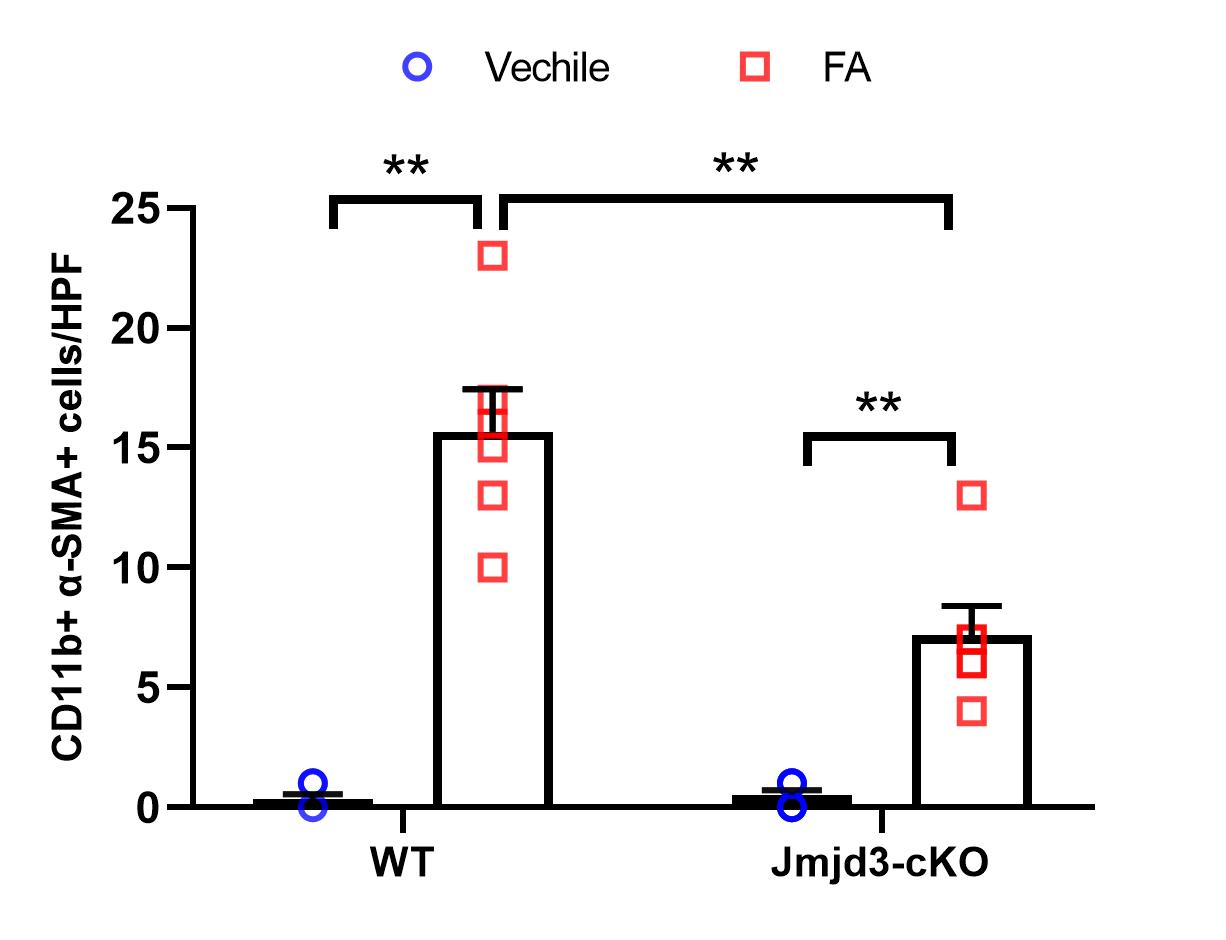

Supplement: Supplementary file 1 [file DataSheet_1.zip › supplementary materials/IF/1-CD11b-SMA-JMJD3KO-FA.jpg]

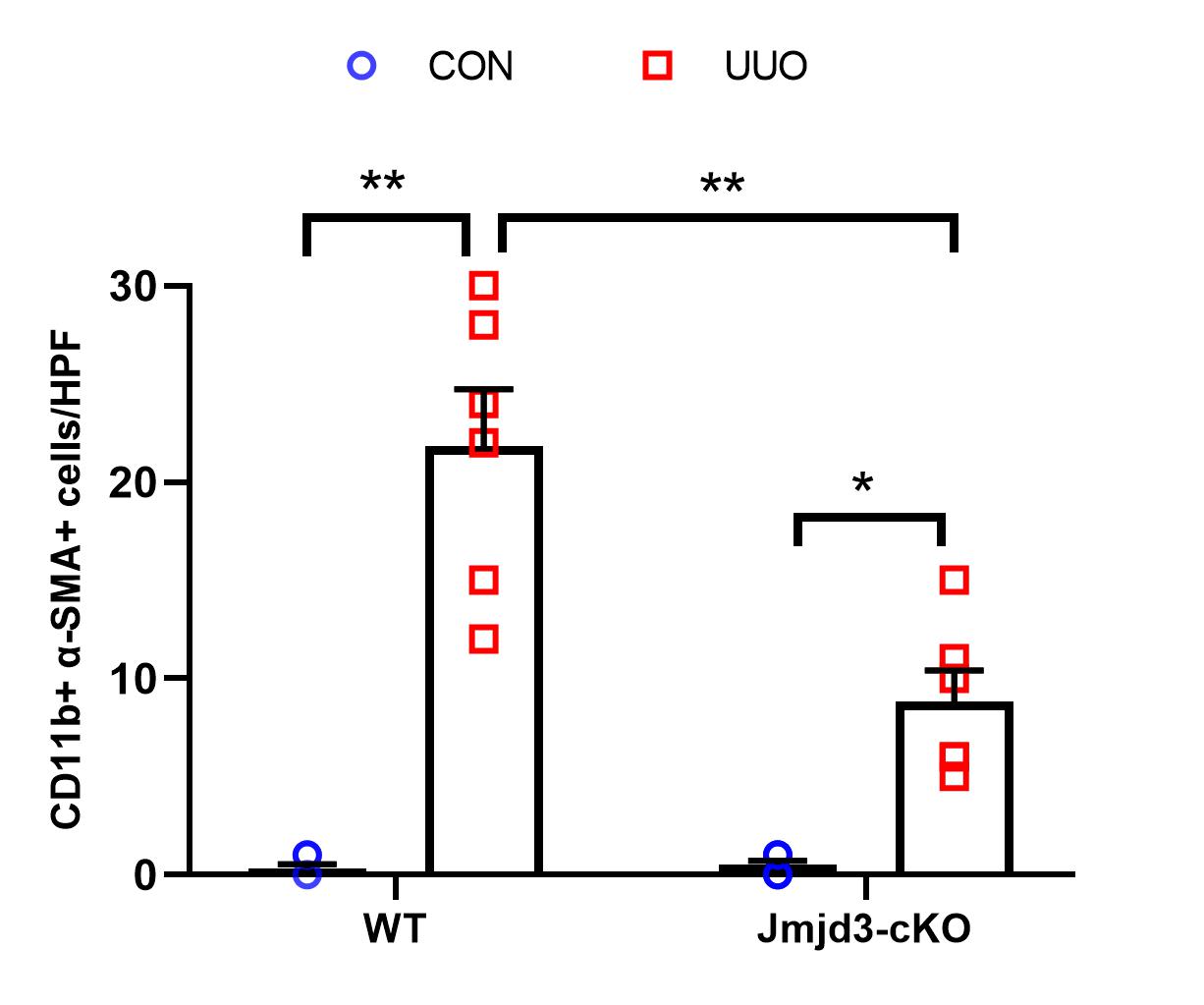

Supplement: Supplementary file 1 [file DataSheet_1.zip › supplementary materials/IF/1-CD11b-SMA-JMJD3KO-UUO.jpg]

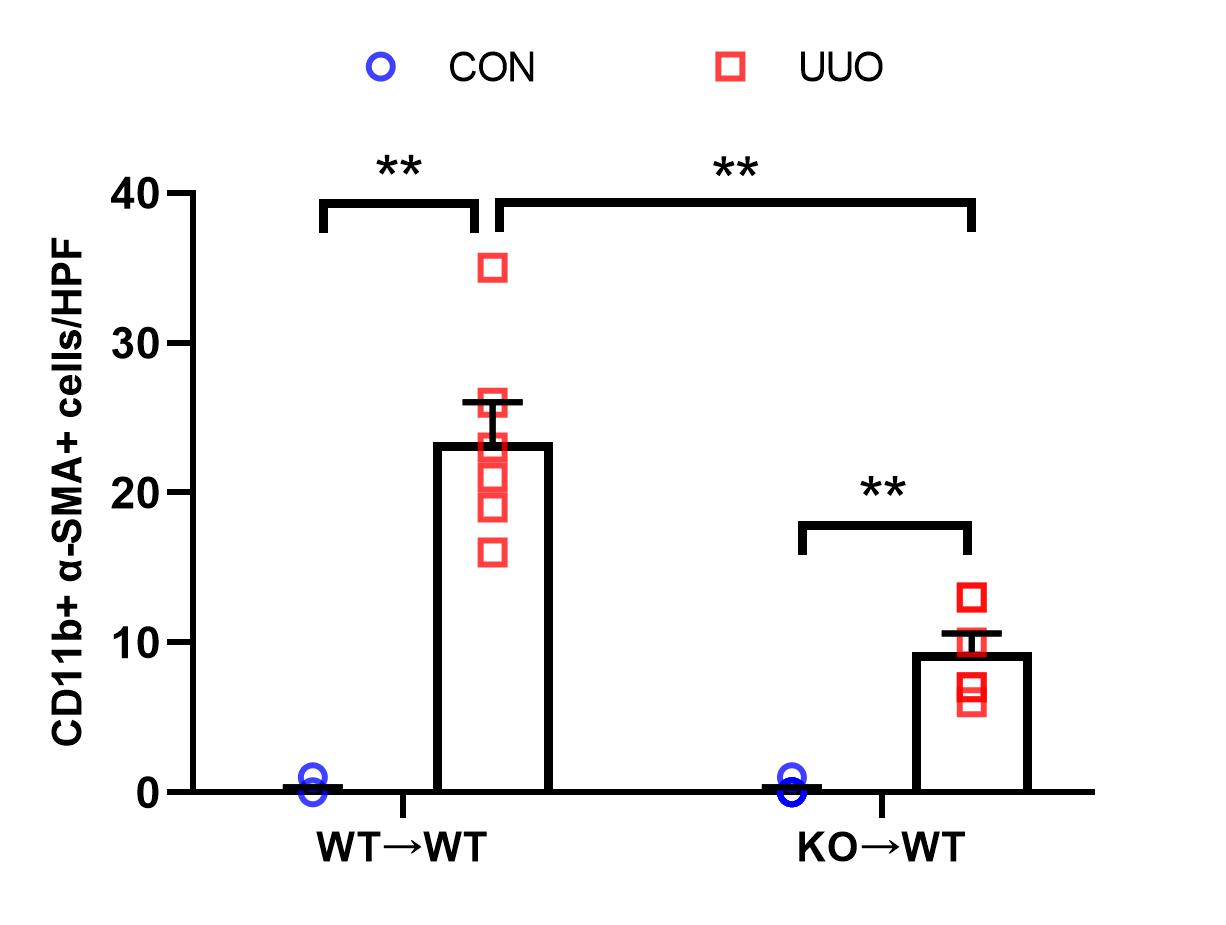

Supplement: Supplementary file 1 [file DataSheet_1.zip › supplementary materials/IF/1-CD11b-SMA-transplantation.jpg]

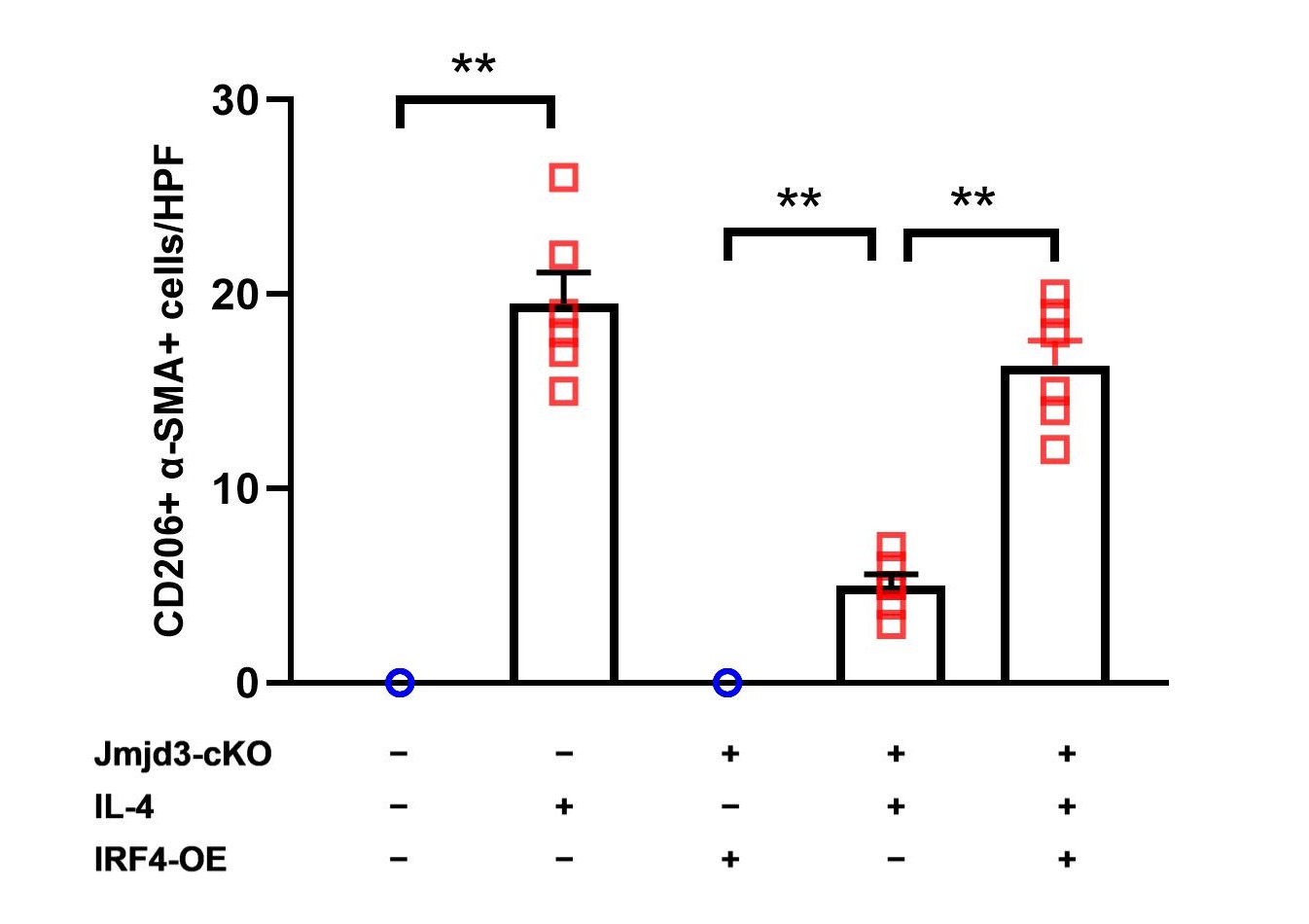

Supplement: Supplementary file 1 [file DataSheet_1.zip › supplementary materials/IF/1-CD206-SMA-JMJD3KO-IL4-IRF4OE1.jpg]

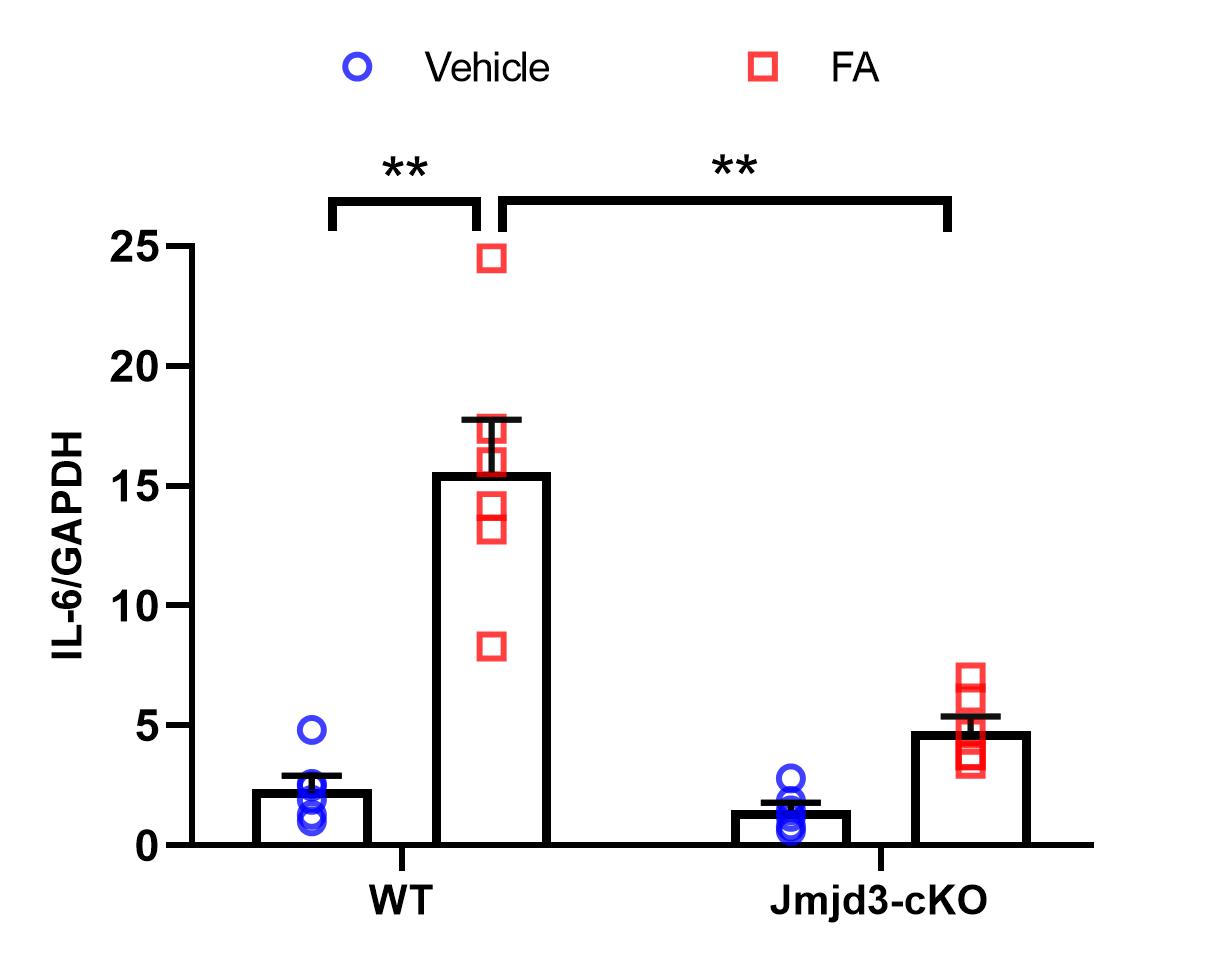

Supplement: Supplementary file 1 [file DataSheet_1.zip › supplementary materials/TNF-a+IL-6/IL-6 PCR.jpg]

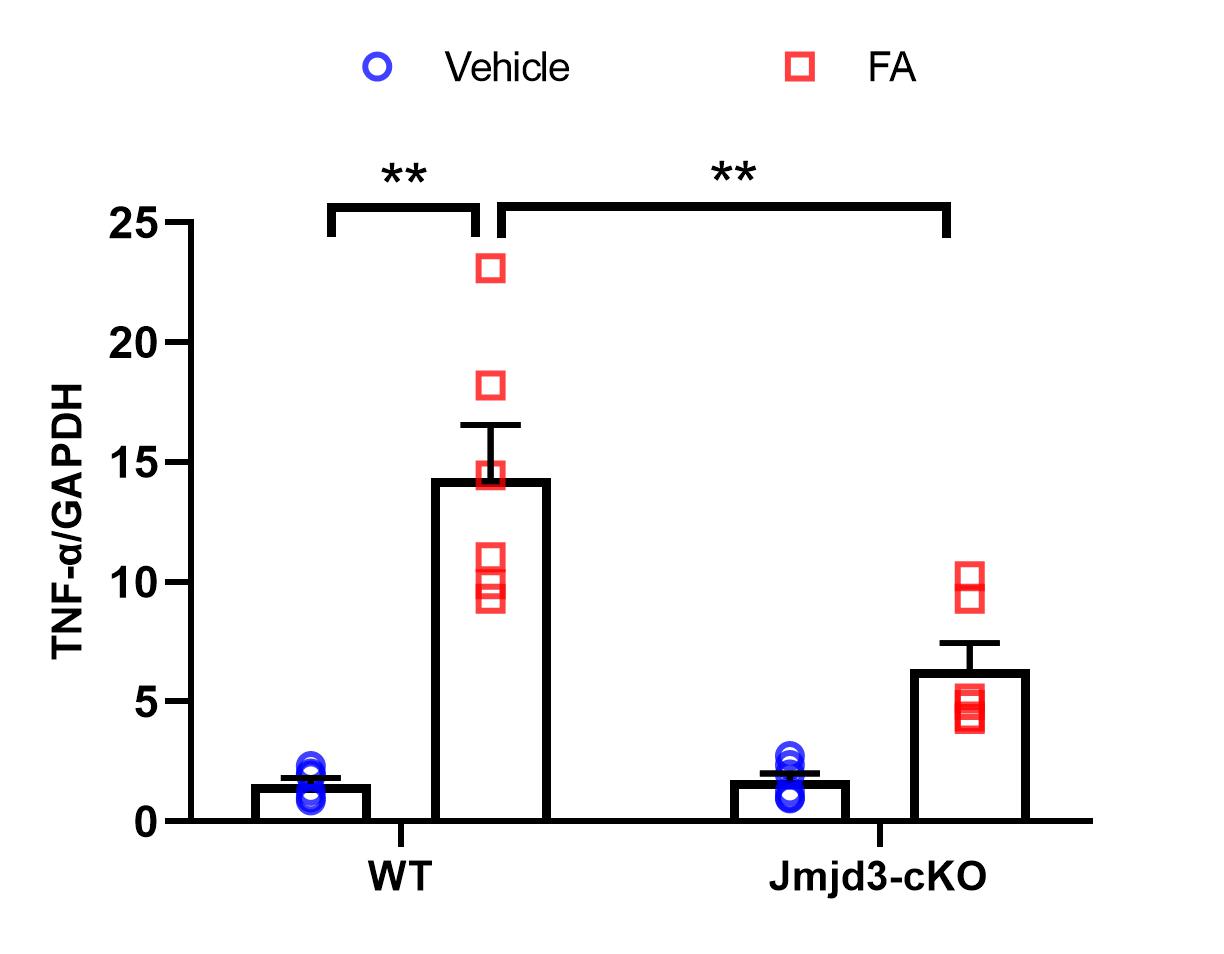

Supplement: Supplementary file 1 [file DataSheet_1.zip › supplementary materials/TNF-a+IL-6/TNFa PCR.jpg]

# Supplementary Figure 1

A

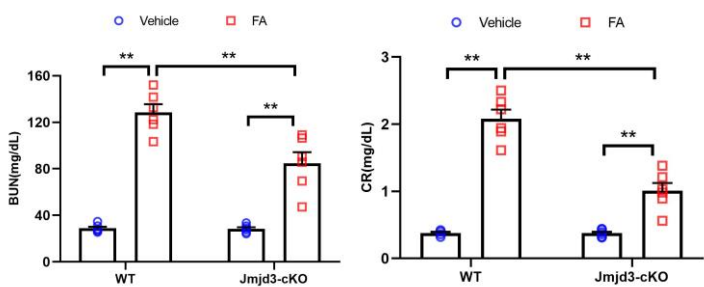

B

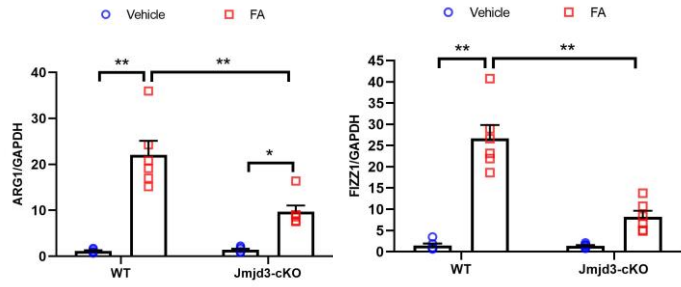

C

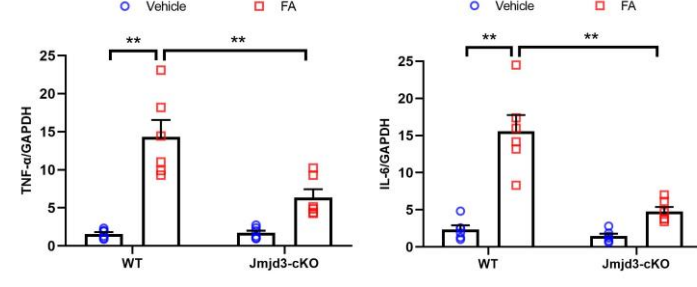

D

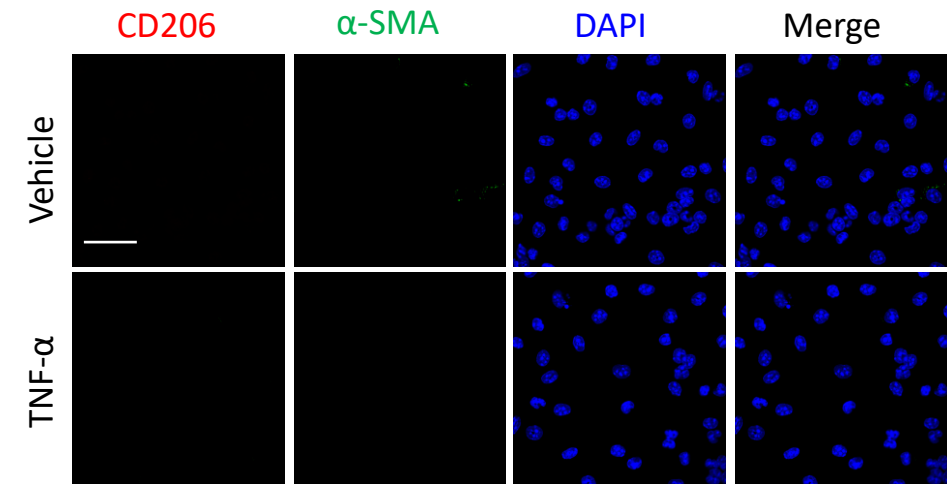

E

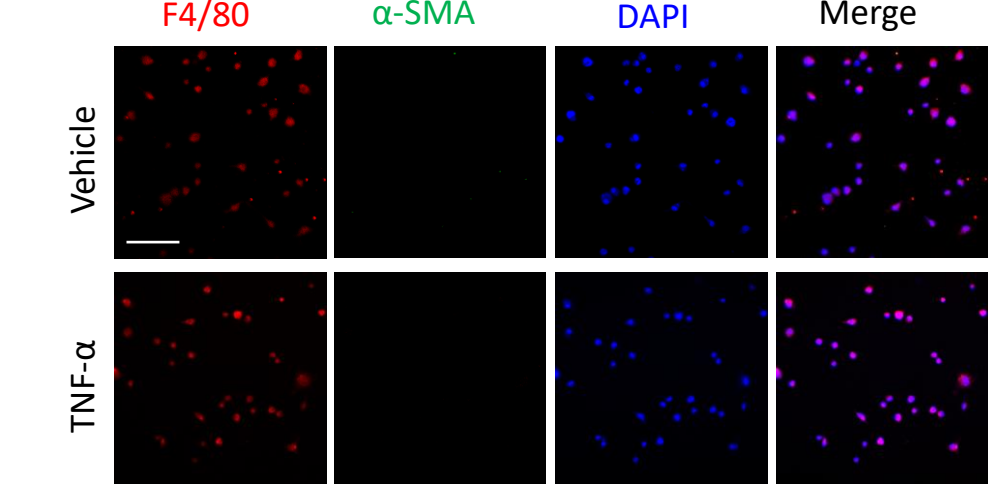

Supplement: Supplementary file 1 [file DataSheet_1.zip › supplementary materials/supplementary figure/supplementary figure 1.pdf]
